# A replicating stem‐like cell that contributes to bone morphogenetic protein 2‐induced heterotopic bone formation

**DOI:** 10.1002/sctm.20-0378

**Published:** 2020-11-27

**Authors:** Julio Mejia, Elizabeth Salisbury, Corinne Sonnet, Zbigniew Gugala, Elizabeth A. Olmsted‐Davis, Alan R. Davis

**Affiliations:** ^1^ Center for Cell and Gene Therapy, Baylor College of Medicine Texas Children's Hospital and Houston Methodist Hospital Houston Texas USA; ^2^ Department of Orthopedic Surgery and Rehabilitation University of Texas Medical Branch Galveston Texas USA; ^3^ Department of Pediatrics—Section Hematology/Oncology Baylor College of Medicine Houston Texas USA; ^4^ Department of Orthopedic Surgery Baylor College of Medicine Houston Texas USA; ^5^ Present address: Methodist Research Institute Houston Texas USA

**Keywords:** adult stem cells, bone, chondrogenesis, Cre‐loxP system, osteoblast, stem cell transplantation, stem/progenitor cell

## Abstract

Bone morphogenetic protein 2 (BMP2)‐induced heterotopic bone formation (HBF) starts synchronously from zero upon BMP2 induction, which is advantageous for lineage tracking. The studies reported here in GLAST‐Cre^Ert2^:tdTomato red (TR)^floxSTOPflox^ mice during BMP2‐induced HBF show 78.8 ± 11.6% of chondrocytes and 86.5 ± 1.9% of osteoblasts are TR^+^ after approximately 1 week. Clustering after single‐cell RNAseq resulted in nine cell types, and analysis revealed one as a highly replicating stem‐like cell (RSC). Pseudotiming suggested that the RSC transitions to a mesenchymal stem‐like cell that simultaneously expresses multiple osteoblast and chondrocyte transcripts (chondro‐osseous progenitor [COP]). RSCs and COPs were isolated using flow cytometry for unique surface markers. Isolated RSCs (GLAST‐TR^+^ Hmmr^+^ Cd200^−^) and COPs (GLAST‐TR^+^ Cd200^+^ Hmmr^−^) were injected into the muscle of mice undergoing HBF. Approximately 9% of the cells in heterotopic bone (HB) in mice receiving RSCs were GLAST‐TR^+^, compared with less than 0.5% of the cells in mice receiving COPs, suggesting that RSCs are many times more potent than COPs. Analysis of donor‐derived TR^+^ RSCs isolated from the engrafted HB showed approximately 50% were COPs and 45% were other cells, presumably mature bone cells, confirming the early nature of the RSCs. We next isolated RSCs from these mice (approximately 300) and injected them into a second animal, with similar findings upon analysis of HBF. Unlike other methodology, single cell RNAseq has the ability to detect rare cell populations such as RSCs. The fact that RSCs can be injected into mice and differentiate suggests their potential utility for tissue regeneration.

## Graphical abstract

Blue arrows show the trajectory of heterotopic bone formation after bone morphogenetic protein 2 induction. The replicating stem‐like cell (RSC) is an epithelial cell that undergoes an epithelial‐mesenchymal transition in forming the chondro‐osseous progenitor (COP) that expresses both osteoblast and chondrocyte transcripts. Red arrows show the fluorescence‐activated cell sorting isolation of either TR^+^ RSCs or TR^+^ COPs and the injection of 3000 of each TR^+^ cell into wild‐type mice undergoing heterotopic ossification.

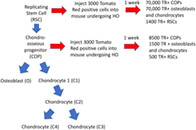

**Significance statement**

Stem/progenitor cells for heterotopic bone have been defined and many found including those as diverse as endothelial and muscle cells. Additionally, the mesenchymal stem cell is thought of as a progenitor for both chondrocytes and osteoblasts. One advantage of single cell RNAseq is that important cells can be identified, even if their numbers are small. In these studies, the authors have found a highly replicating stem‐like cell (RSC) that initiates heterotopic bone formation. The authors have shown that labeled RSCs can replicate and differentiate both in vitro and, when the authors transplant them, in vivo. The RSC is a candidate for bone tissue engineering.

## 1 INTRODUCTION

Bone morphogenetic protein 2 (BMP2) is approved for spinal fusion.^1^ However, several side effects are associated with its use, reducing its application in tissue engineering strategies.^2^ Delivery of this single protein into the muscle leads to the recruitment of all of the necessary stem/progenitor cells to create new vascularized innervated bone,^3^, ^4^ and therefore remains a promising therapeutic strategy. To date several groups have characterized potential stem/progenitors involved in heterotopic bone formation (HBF). This includes the identification of vascular endothelial cells^5^ as well as skeletal muscle stem cells^6^ as osteoblast precursors. There has also been a periosteal stem cell identified that expresses both osteoblast and chondrocyte transcripts and is responsible for intramembranous bone formation,^7^ as well as a leptin‐expressing mesenchymal stem cell.^8^ A growing consensus of potential stem/progenitors cells for HBF is that they are derived from local tissues and express platelet‐derived growth factor receptor alfa (PDGFRα).^7^, ^9^, ^10^ However, PDGFRα‐expressing progenitors have not been isolated and definitively characterized to show their similarities to or differences from other reported skeletal progenitors.

Glial high affinity glutamate transporter (GLAST) was first reported in osteoblasts and osteocytes in bone by Mason et al in 1997^11^ when it was noted to be expressed in newly formed bone after loading, but not in quiescent bone. Recently, Kan et al^12^, ^13^ reported several types of chondro‐osseous progenitors that express GLAST (or Slc1a3). Kan et al^13^ also reported that these progenitors express glial fibrillary acidic protein (GFAP), which is highly specific for astrocytes.^14^ However, bone formation occurred normally in mice possessing constructs in which GLAST or GFAP promoters drove expression of diphtheria toxin A,^13^ leaving doubt whether these cells were true progenitors. Further adding to the confusion, Kan et al reported that GLAST was found in mature chondrocytes associated with heterotopic bone and was not present on skeletal cartilage.^12^ We have found that some commercially available antibodies for GLAST exhibit poor binding ability. In our hands, there has sometimes been poor correlation between reporter expression and antibody binding; therefore, we used the GLAST‐Cre^Ert2^:tdTomato Red (TR)^floxSTOPflox^ model in order to identify the progenitor cell population responsible for HBF.

Surprisingly, preliminary studies using this GLAST‐Cre^Ert2^:tdTomato Red (TR)^floxSTOPflox^ model revealed that the heterotopic as well as the skeletal bone of these mice had no cells that were TR^+^ when these mice were treated with tamoxifen alone, whereas a high percentage of cells in heterotopic bone were formed and labeled in the presence of both tamoxifen and BMP2. Here, we present studies that confirm through single‐cell RNA sequencing the presence of GLAST expression in early chondro‐osseous stem/progenitor cells and demonstrate their ability to traffic through a mesenchymal stem‐like cell (MSC) intermediate state to form bone and cartilage. We suggest that the earliest cell in this trajectory goes through an epithelial to mesenchymal transition (EMT) like neural crest stem cells. Surprisingly, the earliest cell was found to have only a few surface markers and be highly replicating, which allowed for culturing. Overall, this study identifies an earlier stem/progenitor cell involved in de novo bone formation, which may be a promising addition for musculoskeletal tissue engineering.

## 2 MATERIALS AND METHODS

### 2.1 Cell culture

Replicating stem‐like cells (RSCs), chondro‐osseous progenitors (COPs), and W20‐17 cells (bone marrow mesenchymal cell line) were plated in 10% fetal bovine serum (FBS) (Corning Cat# 35‐010‐CV) in DMEM supplemented with 1× antibiotic‐antimycotic solution (Gibco Cat# 15240‐062, Proliferation media) at a density of approximately 550 cells/cm^2^ and allowed to replicate until approximately 80% confluence. Osteogenesis: Cells at 80% confluence were placed in osteogenic media (αMEM media supplemented with 10% FBS, 1× antibiotic‐antimycotic, 200 nM ascorbic acid, and 10 mM glycerol 2‐phosphate). Adipogenesis: Cells at 80% confluence were placed in Mesencult Media (Cat #05507) containing a proprietary supplement, 1× antibiotic‐antimycotic, and 2 mM l‐glutamine. Chondrogenesis: Cells were removed from dishes with 0.25% trypsin, pelleted at 400*g* for 5 minutes, and pelleted cells with residual media placed in a tissue culture dish and allowed to adhere for 3 hours in a tissue culture incubator at 37°C. The cultures were then supplemented with chondrogenic media (αMEM supplemented with 10% FBS, 1× antibiotic‐antimycotic, and 10 ng/mL rBMP2). A subset of cells remained in their original media. Of note, the COP cells did not replicate well, therefore only 52 000 cells were present in a chondrogenic pellet, but there were three replicates. Alternatively, the RSCs replicated to confluence and chondrogenic pellets were established using 520 000 cells per pellet. This may be a factor in the cell death observed in the COP cells. For osteogenesis the cells were stained with alizarin red (Alizarin red stain kit, IHC World, catalog # IW‐3001). For adipogenesis the cells were stained with oil red O (Oil red O stain kit, IHC World, catalog #IW‐3008). Finally, for chondrogenesis the cells were stained with Alcian blue and counterstained with nuclear red using a kit (IHC World, catalog # IW‐3000) in accordance with the manufacturer's instructions.

### 2.2 Lineage tracking

Ert2 (tamoxifen inducible) GLASTCre transgenic mice were obtained from Jackson Laboratory (Stock No. 12586). These mice were crossed with the R26R td Tomato red mouse (Stock No. 007914, Jackson Laboratory), which contains a constitutive promoter, an intervening sequence flanked by lox P sites, and the fluorescent reporter td Tomato red (TR)^15^ to generate GlastCre^Ert2^:tdTR^floxSTOPflox^ mice. The mice received tamoxifen (free base), 1 mg per mouse, (Sigma‐Aldrich, St. Louis, Missouri) or vehicle control (9:1 [vol/vol], sunflower oil: 100% ethanol) through three consecutive intraperitoneal injections at the indicated times. Wild‐type mice refer to C57BL/6 that is the background strain for the reporter mice.

### 2.3 Induction of HBF

Replication‐defective E1‐E3‐deleted human type 5 adenovirus possessing cDNA for BMP2 (AdBMP2) in region E1 was constructed as previously described.^3^ Mouse skin fibroblasts were transduced at 5000 virus particles per cell with 0.75% GeneJammer to achieve greater than 90% transduction efficiency as described previously.^16^ All adenoviruses were negative for replication competent adenovirus. AdBMP2‐transduced cells (5 × 10^6^ cells) were resuspended in saline and injected into the hind limb muscles. Transduced cells (5 × 10^6^) were confirmed to express approximately 20 ng of BMP2. All studies involving recombinant DNA followed the NIH guidelines.

### 2.4 Flow cytometry and fluorescence‐activated cell sorting

The cells from the hind limb tissues were isolated and digested with collagenase type 2 as previously described.^17^ Briefly, hind limb muscle tissue was dissected from the skeletal bone into cold Hanks buffered saline solution (HBSS) and dissociated by mincing the tissues and incubating for 45 minutes at 37°C in 0.2% type 2 collagenase (Worthington) in HBSS. An equal volume of Dulbecco's modified Eagle's medium supplemented with 10% fetal bovine serum was added to quench the digestion reaction. Dissociated cells were centrifuged, triturated, filtered through nylon mesh and resuspended in cell staining buffer. Fluorescence‐activated cell sorting (FACS) was performed using a FACS Aria II cell sorter (BD Biosciences, San Jose, California) equipped with analyzing software (BD FACSDiva software version 8.0.1, BD Biosciences) for the red reporter. Cells were incubated with several antibodies (Hmmr (Abcam polyclonal antibody, ab124729) and Cd200 (Invitrogen, rat monoclonal antibody, MA1‐70101)) and then visualized using Alexa Fluor secondary antibodies (1:500 dilution; 488, 594, or 647; Invitrogen Life Technologies, Carlsbad, California). For cell sorting, labeled cells were separated based on their fluorescence intensity and all populations collected with 95% purity.

### 2.5 Serial transplantation

To obtain RSC and COP cells for serial transplantation, HBF was induced in GLAST‐TR^+^ transgenic mice receiving daily tamoxifen injections. Five days after induction of HBF, GLAST‐TR^+^ cells were isolated as described in the previous section. All samples were then stained with Hmmr and CD200. GLAST‐TR^+^ Hmmr^+^ Cd200^−^ cells (RSC) and GLAST‐TR^+^ Cd200^+^ Hmmr^−^ cells (COP) were sorted from 3 mice for either the RSC or COP preparation and pooled. Each group of cells were then spun down at 500*g* 10 minutes and resuspended in pharmaceutical grade saline. Approximately 3000 RSC or COP cells were injected into the quadriceps muscles of 6 C57BL/6 mice that had undergone HBF for 4 days prior to transplantation. These transplanted mice were euthanized after 4 (RSC) or 10 (COP) additional days without any further treatments, and tissues/cells were isolated, stained, analyzed, and sorted in the same manner described above. However, not enough COP cells could be sorted from the transplanted mice and thus this group had to be dropped from the second‐round transplantation. The collected RSCs were then prepared and transplanted at 600 cells/mouse into the quadriceps of another set of 6 C57BL/6 mice that had undergone HBF for 4 days prior to transplantation, and 7 days post‐transplantation tissues/cells were isolated, stained, and analyzed in the same manner as described above.

### 2.6 Single‐cell RNAseq

GLAST‐Cre^Ert2^:TR^floxSTOPflox^ mice induced with BMP2 on day 0 were pulsed with tamoxifen every day for 10 days, and GLAST‐TR^+^ cells were isolated by FACS. Approximately 5000 cells were resuspended at 500 cells/μL and provided to the BCM Single Cell Genomics core facility at our institution. An estimated 3228 cells were successfully processed and coded using a 10× Genomics platform (10× Genomics, Pleasanton, California) and a library was prepared according to the manufacturer's instructions (10× Genomics). The library was sequenced to a depth of 143 000 reads per cell (Genewiz, Summerfield, New Jersey). Data were processed through a Cell Ranger pipeline (10× Genomics), converted to a sparse matrix and then clustered using the Seurat (version 3) algorithm.^18^ The Seurat version 3 algorithm can easily separate cell types because it utilizes the mathematically‐derived Uniform Manifold Approximation and Projection (UMAP) rather than t‐SNE as the dimension reduction technique for machine learning.^19^ All data were processed using the R and R Studio software packages (Boston, Massachusetts). Cell cycle regression was run to check for clustering due to the cell cycle, and we found no evidence for clustering due to the cell cycle (data not shown). Pseudotiming of the data was performed using the Monocle (version 2) algorithm.^20^ To convert to Monocle, a Seurat (version 3) object was converted to a Monocle Cell Data Set file. In order to confirm the trajectory depicted by the Monocle algorithm, each branchpoint was subjected to Branched Expression Analysis Modeling (BEAM).^21^

### 2.7 Immunohistochemistry

Immunohistochemistry was performed as previously described.^22^ Tissues were isolated and treated with sucrose to preserve the fluorescent reporter prior to snap freezing and sectioning. Serial sectioning was performed at a depth of 2‐4 μm. Primary antibodies were used at a dilution of 1:100 to 1:200, and secondary antibodies (Alexa Fluor 488, 594, or 647; Invitrogen Life Technologies, Carlsbad, California) at a 1:500 dilution. Primary antibodies used were as follows: Sp7 (Santa Cruz Biotechnology, Inc, Dallas, Texas, monoclonal sc‐393 325), Sox 9 (Abcam, monoclonal, ab185966), Birc5 (Abcam, polyclonal, ab469), and Ki67 (Abcam, ab15580).

Primary and secondary antibodies were diluted in PBS with 2% bovine serum albumin. Tissues were counterstained and covered with Vectashield mounting medium containing DAPI (Vector Laboratories, Burlingame, California) and when DAPI was absent, with Prolong Antifade (Invitrogen Life Technologies). Stained tissue sections were examined using an Olympus BX41 microscope (Olympus Corporation of the Americas, Waltham, Massachusetts) equipped with a reflected fluorescence system. To ensure signal specificity, controls were performed and the specific absorption spectrum from each primary‐secondary pair was captured.

### 2.8 Quantification

For quantification and to ensure representative sections, the entire limb was sectioned serially at a depth of 20 μm. To locate the region of HBF, every fifth slide was stained with hematoxylin (Harris Hematoxylin, American Mastertech, Lodi, California) and eosin (Eosin Y Phyloxine B solution, Electron Microscopy Sciences, Hatfield, Pennsylvania). The entire region of HBF was localized and sections within the center of the lesion were selected as representative. Hematoxylin and eosin serial sections were captured by bright field microscopy using the Olympus BX41 scope. For immunohistochemistry, the images were captured using a Cytation 5 (Biotek Instruments, Winooski, Vermont) imaging reader digital confocal microscope that can reconstruct the image, and the Fiji version of Image J was used to calculate the percentage of chondrocytes (Sox9^+^ or aggrecan^+^ cells) or osteoblasts (Sp7^+^ or osteocalcin^+^ cells) that also co‐express GLAST‐TR using the Fiji plugin (Coloc2) for colocalization analysis. Tissues were co‐stained using Dapi. A complete tissue section was counted entirely to gather a single percentage point and a minimum of six sections from different mice were counted to calculate the mean. SE of the mean was used to determine error.

## 3 RESULTS

### 3.1 GLAST is expressed during BMP2‐induced HBF

**FIGURE 1:**
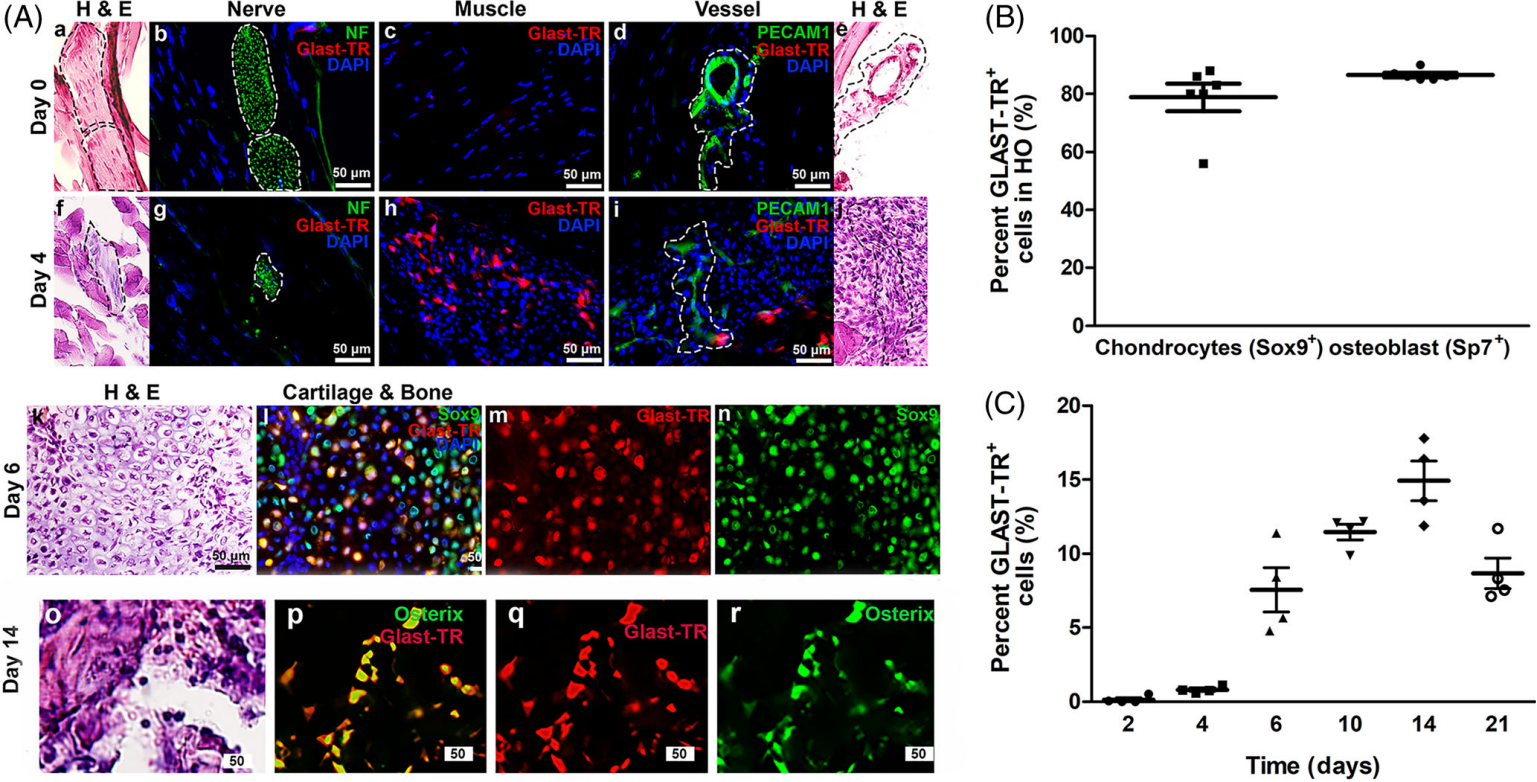
GLAST‐TR is expressed in chondrocytes and osteoblasts during HBF. A, Representative photomicrographs of GLAST‐TR^+^ expression in tissue during HBF. GlastTR, Neurofilament H (NF), and platelet‐endothelial cell adhesion molecule 1 (Pecam, Cd31) were detected through immunohistochemical staining during HBF and are depicted for days 0 (panels a–e) and 4 after induction with GLAST‐TR^+^ cells first observed at day 4 (panels f–j). In addition, GLAST‐TR^+^ Sox 9^+^ chondrocytes were observed in newly forming cartilage in tissues isolated 6 days after induction of bone formation (panels k–n). Furthermore, by day 14, GLAST‐TR^+^ Sp7^+^ osteoblasts were observed associated with bone matrix (panels o–r). B, Tissues undergoing cartilage formation 6 days after BMP2 induction in the presence of tamoxifen were analyzed and Sox9^+^ chondrocytes and Sox9^+^ GLAST‐TR^+^ dual positive chondrocytes from six samples were quantified using image J. The resultant percentages of GLAST‐TR^+^ chondrocytes (Sox9^+^) are depicted graphically (symbols), the horizontal bar and error bars represent the group mean and SEM, respectively. Similarly, the percentage of GLAST‐TR^+^ osteoblasts was calculated from six samples. Osteoblasts were defined as Sp7^+^ cells associated with bone matrix. Symbols on the graph are actual data points and the horizontal line depicts the group mean. The error bars represent SEM. C, Results of flow cytometry analysis to quantify the number of GLAST‐TR^+^ cells in the hind limb. Symbols are the actual data points and a horizontal line represents the mean per group. The vertical line depicts the SEM

Following induction with BMP2, HBF was established in GLAST‐Cre^Ert2^:tdTR^floxSTOPflox^ reporter mice. Mice induced for HBF were injected with tamoxifen on days −1, 0, 1, and 2. We isolated and analyzed tissues for the presence of the tomato red (TR) reporter. We did not observe GLAST‐TR^+^ cells in the tissues on day 0 (Figure 1A, panels a–e); however, we detected them as early as 4 days after induction. GLAST‐TR^+^ cells were not associated with vessels (CD31^+^ endothelial cells, green color) or nerves (neurofilament heavy chain^+^ nerves, green color), but were found in muscle surrounding the site of HBF (Figure 1A, panels f to j). At later times, GLAST‐TR^+^ cells were found to be associated with cartilage (Sox 9^+^ cells, green, Figure 1A, panels k to n) and bone (Sp7^+^ cells, green, Figure 1A, panels o to r). The GLAST‐TR^+^ Sox9^+^ dual positive cells and Sox9^+^ total chondrocytes were quantified using image J from tissues undergoing HBF for 6 days. The percentage of GLAST‐TR^+^ cells associated with each section is depicted (Figure 1B) with a mean of 78.8 ± 11.6% of Sox9^+^ chondrocytes^23^ that are GLAST‐TR^+^ (Figure 1B). Similarly, we quantified the number of GLAST‐TR^+^ Sp7^+^ dual positive and total Sp7^+^ cells in tissues isolated 14 days after induction of HBF (Figure 1B). The percentage of GLAST‐TR^+^ osteoblasts (Sp7^+^ cells) was then calculated and depicted graphically (Figure 1B). Approximately 86.5 ± 1.9% of all the osteoblasts as determined by positive expression of Sp7^24^ were positive for GLAST‐TR (Figure 1B).

Quantification by flow cytometry of the GLAST‐TR^+^ cells within hind limb soft tissues showed a continuous increase in their number over a 2‐week period until they reached a maximum of approximately 15% of total cells (Figure 1C). No GLAST‐TR^+^ cells were identified in the tissues prior to delivery of BMP2, yet a small but distinct population was observed 2 days after induction (Figure 1C), but these were not associated with any specific structures in the limb (data not shown). We established the flow cytometry gates using similar mice undergoing bone formation without tamoxifen. These mice did not express the reporter (Supporting Information Figure S4). The number of GLAST‐TR^+^ cells peaked at day 14, then dropped slightly by day 21 (Figure 1C), likely due to remodeling of the heterotopic bone, to a more compact structure, like fracture callus.

### 3.2 GLAST‐TR^+^ cells are labeled during the first 48 hours of HBF

To determine the timing of GLAST expression and rearrangement of the GtROSA26Sor locus during BMP2‐induced HBF, we reduced tamoxifen treatment to short pulses by varying its time of delivery and withdrawal.

**FIGURE 2:**
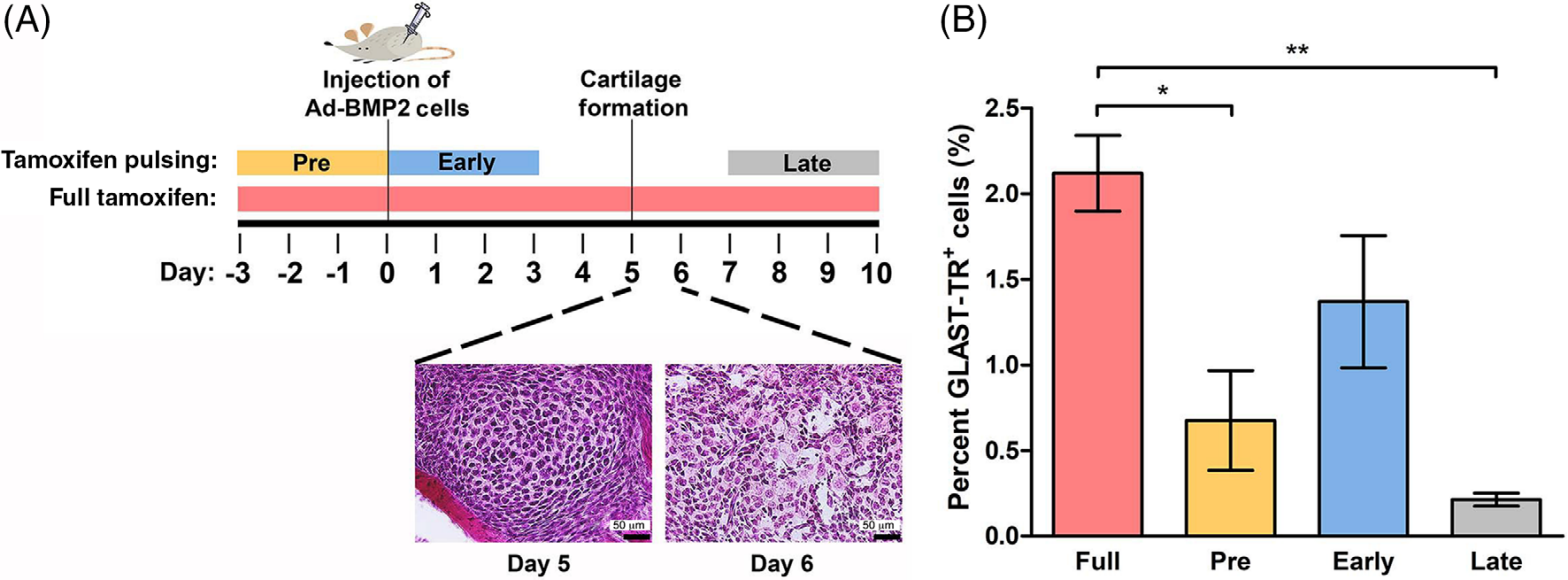
The majority of GLAST‐TR^+^ cells are labeled within 48 hours after delivery of BMP2. A, Schematic timeline of tamoxifen pulses, with respect to both delivery of BMP2 and the appearance of cartilage within the tissues. B, Graphical depiction of the percent of GLAST‐TR^+^ cells within the tissues at 10 days after various tamoxifen pulses as determined by flow cytometry. Bars are the average of six mice. The vertical line depicts the SEM. Asterisk and horizontal bars represent statistically significant differences. *P < .05; **P < .01

We established three pulse periods during HBF. The first pulse was conducted just prior to induction of HBF, thus labeling pre‐existing cells expressing GLAST in the mouse (Pre) (Figure 2A). The second pulse period consisted of delivering tamoxifen at the same time as induction of HBF (Early) to label cells that might be induced directly by BMP2 to express GLAST. The final pulse tested (Late) coincided with the initial appearance of chondrocytes and osteoblasts within the tissues (Figure 2A) and after the termination of BMP2 expression.^3^, ^25^ As a control, we also delivered tamoxifen daily throughout the entire experimental period (Full) to provide an estimate of the total number of GLAST‐TR^+^ cells that could be labeled in the mice (Figure 2A). We determined by flow cytometry the number of GLAST‐TR^+^ cells labeled during each period (Figure 2B). Comparison between groups suggested a statistically significant difference between the Full pulse and either the Pre or Late pulse (Figure 2B). However, there was no statistically significant difference between the Early and Full groups (Figure 2B), suggesting that cells become GLAST^+^ early in the formation of HB.

In all cases, heterotopic bone was formed similarly, indicating the amount of bone does not account for the variable numbers of GLAST‐TR^+^ cells quantified by flow cytometry (data not shown). Analysis of the heterotopic bone showed varying numbers of osteoblasts and chondrocytes expressing TR depending on the tamoxifen pulse with a striking reduction in GLAST‐TR^+^ osteoblasts and chondrocytes in the late pulse (data not shown). Overall, these results suggest that osteoblasts and chondrocytes express TR only if the GLAST promoter is activated earlier in their lineage, within the first 48 hours after BMP2 delivery.

### 3.3 Single‐cell RNAseq and clustering of isolated GLAST‐TR^+^ cells reveals RSCs and COPs that emerge during HBF

**FIGURE 3:**
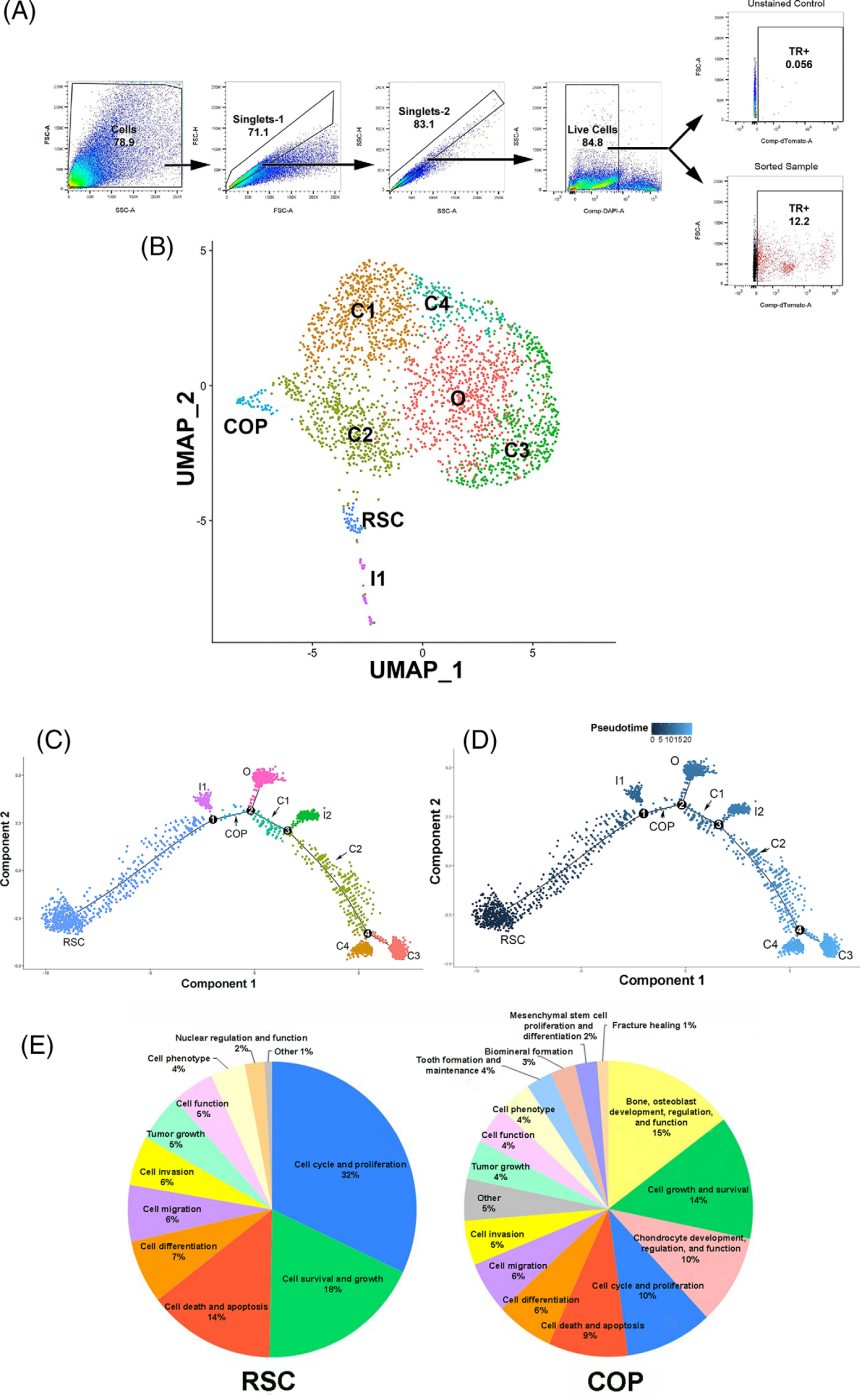
Single cell transcriptome analysis of isolated GLAST‐TR^+^ cells after induction of HBF. A, Flow cytometry gating strategy for isolation of the GLAST‐TR^+^ cells used for single‐cell RNA seq. B, Single‐cell RNA seq clusters 0 to 8 were determined by analyzing the GLAST‐TR^+^ single‐cell RNAseq data using the Seurat (v3) algorithm. The tentative contaminating cluster I2 is not shown and fell between 5‐10 UMAP1 and 2 UMAP2 units. C, A trajectory was plotted using Monocle 2. D, Pseudotiming of the clusters was performed using the Monocle 2 algorithm and shows the relationship of the cells to one another in pseudotime. Numbers depict potential branchpoints and lines suggest the cells are related. E, Pie charts depict the percentage of transcripts in the stem‐like cell (RSC) and chondro‐osseous progenitor (COP) transcriptomes in various categories as determined using Pathway Studio

Ten days after induction of HBF, we isolated by flow cytometry GLAST‐TR^+^ cells from tissues in and around the site of bone formation (Figure 3A) to ensure the presence within the sample of all stages of cells, from progenitor to mature osteoblasts and chondrocytes. We then conducted single‐cell RNA seq and analyzed the resultant transcriptome data using the Seurat (v3) algorithm^18^ that clusters similar cells into distinct groups. This analysis yielded nine clusters (Figure 3B) with a transcript list for each cluster (Supporting Information Table S1 shows the first 50 transcripts in each cluster). Using the transcriptomes of each cluster, we performed Gene Ontology (GO) analysis (Supporting Information Table S2). The results allowed a cell‐type designation to be assigned to each cluster, except two clusters; I2 was most likely a contaminant and thus is not further depicted while the other, (I1), remains unknown. Each of the remaining seven clusters expressed transcripts for the rearranged Gt(Rosa)26Sor locus (Supporting Information Table S3), while expression of the GLAST transcript was present in all but cluster 7 (I1).

Replicating stem‐like cells (RSCs) possess many transcripts associated with replication (Figure 3E, Supporting Information Table S1). We adapted the RSC designation because of the transcriptome analysis in Pathway Studio (Elsevier, Amsterdam), which suggested this population most closely resembled a cancer stem cell. In addition, the RSC transcriptome strongly resembles a discrete stage of a neural stem cell (activated [early‐middle] NSC), which represents the replicative stage of the life cycle of the NSC as defined by Dulken et al.^26^ Thus, the transcriptome of RSCs suggests that they may be replicating stem‐like cells.

Transcriptome analysis also revealed one cluster of GLAST‐TR^+^ cells expressed both chondrocyte‐ and osteoblast‐specific transcripts, and we have termed these chondro‐osseous progenitors (COPs). Surprisingly, while these cells possess early osteogenic (Sp7) and chondrogenic (Sox9) transcripts, they also simultaneously express late osteogenic (Osteocalcin) and chondrogenic (Aggrecan) transcripts. However, the recently described periosteal stem cell^7^ appears to have a similar transcriptome. GO analysis was unable to categorize this cell, but analysis in Pathway Studio revealed them to be COPs (Supporting Information Table S2).

We also analyzed the transcriptomes of each cluster for genes associated with chondrocytes and osteoblasts (Supporting Information Table S4). Some transcripts such as S100a11^27^ have been associated with chondrocyte function, but are not chondrocyte specific and in this case were also observed in COP and RSC clusters. Also, some osteoblast associated but not specific transcripts, such as Sparc (osteonectin)^28^ were present in other clusters such as COP and RSC. However, other transcripts such as Gnas (Gsα) ^29^ and Igf1^30^ are essential for osteogenesis and expressed highly in osteoblasts. Likewise both Matn4^31^ and Aspn^32^ have been shown to be important in chondrogenesis, and both are highest in specific chondrocytes, C1 and/or C2. Beyond the COP cluster, GO analysis identified four clusters that are like chondrocytes and one osteoblast‐like cluster.

The data were analyzed using the Monocle algorithm,^20^ which plots the order of cell types and their relationship to one another in their differentiation pathway called pseudotime.^20^, ^33^, ^34^, ^35^ Results of this analysis show a trajectory in which all clusters are connected, suggesting that they are associated and may represent different functional or differentiation states of a common cell type (Figures 3C). The plot in pseudotime shows the RSC to be the earliest cell type, which then transitions to the COP (Figure 3D).

We used Pathway Studio to analyze the transcripts for each of these clusters and found that RSCs have approximately four transcripts that would be membrane associated and/or surface markers. This is extremely unique for a cell population. Furthermore, approximately 32% of its transcriptome is involved in cellular replication, 18% in cell survival and growth, and 14% in regulating cell death. Alternatively, the COP has a more uniform distribution of surface markers and a reduced number of transcripts involved in replication (10%), suggesting a transition from a replicating cell to a cell undergoing chondro‐osseous differentiation, with 2% of its transcriptome expressing transcripts involved in mesenchymal differentiation, osteogenesis (15%), and chondrogenesis (10%) (Figure 3E).

### 3.4 The RSC and COP are present during HBF

**FIGURE 4:**
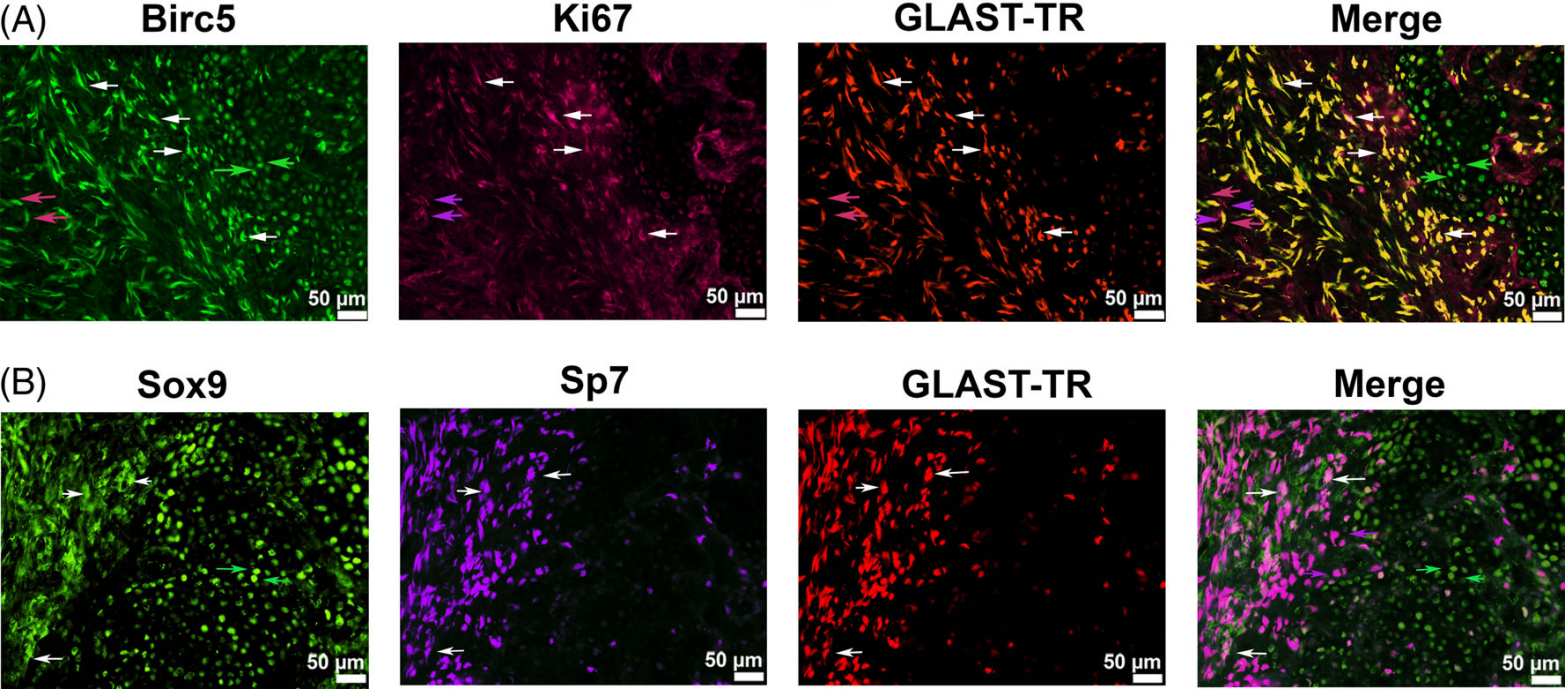
Representative photomicrographs of immunostaining confirming the existence of RSCs and COPs in tissues isolated from HBF. Tissues were isolated 10 days after BMP2‐induced HBF, fixed using sucrose, snap frozen, serial sectioned, and immunostained. A, Representative photomicrograph of Ki67(magenta), GLAST‐TR (red), Birc5 (green). White arrows show triple positive cells; green arrows show cells positive only for Birc5; red arrows show cells positive only for Glast‐TR and Birc5; purple arrows show cells positive only for Ki67. B, Representative photomicrograph of Sox9 (green), GLAST‐TR (red), Sp7 (magenta), and the merge. White arrows show triple positive cells; green arrows show cells positive only for Sox9; magenta arrows show cells positive only for Glast‐TR and Sp7

To confirm the presence of the RSC and COP in vivo, tissues were isolated during HBF and analyzed for the presence of GLAST‐TR^+^ cells specific for the RSC (Ki‐67^+^ and Birc5^+^ [survivin]) or the COP (Sox9^+^ and Sp7^+^ [osterix]). These cell‐specific markers were selected based on the transcriptomes (Supporting Information Table S1). Both GLAST‐TR^+^ Ki‐67^+^ Birc5^+^ and GLAST‐TR^+^ Sox9^+^ Sp7^+^ cells were identified in tissue isolated 10 days after induction of HBF (Figure 4A,B, respectively), confirming the presence of these two populations in vivo during HBF (Figure 4A,B). We found these cells are present as early as 6 days after induction and persist even after BMP2 is removed and bone formation has peaked, suggesting their stem‐like cell nature. Among cells positive for the RSC, we also found cells positive for only Birc5 (green arrows, Figure 4A), for TR and Ki67 (red arrows, Figure 4A), and cells positive for only Ki67 (magenta arrows, Figure 4A). Likewise, among the cells positive for the COP, we also found cells positive for only Sox9 (green arrows, Figure 4B) as well as cells positive for TR and Sp7 (magenta arrows, Figure 4B). The data confirm expression of key transcripts in the RSC and COP clusters.

### 3.5 BEAM for ordering the clusters suggests the RSC transitions to the COP through an EMT

**FIGURE 5:**
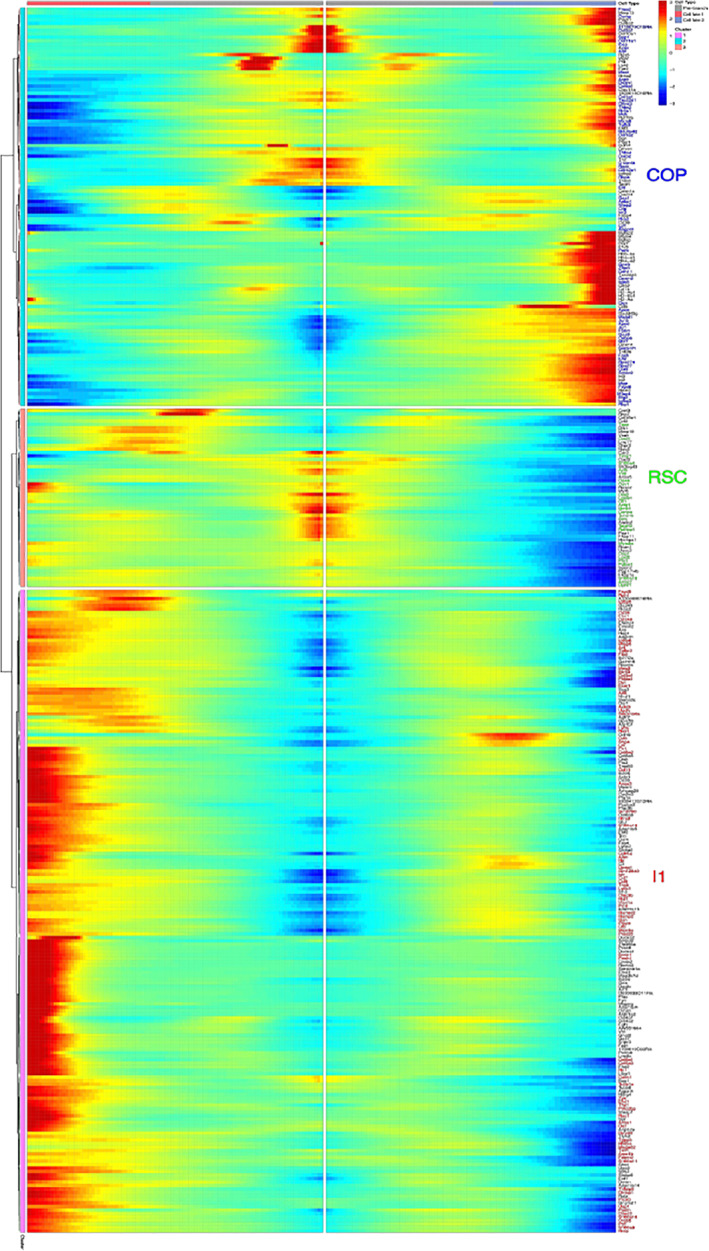
Branched Expression Analysis Modeling (BEAM) for analysis of Branchpoint 1. All scRNAseq data was analyzed using Monocle 2. The first branchpoint is depicted, which shows that the transcripts fall either into the transition state (gray zone of top bar, zoom to enlarge), cell fate 1 (blue zone), or cell fate 2 (red zone). See Supporting Information Figure S1 for other branchpoints. BEAM analysis depicts the transcripts (shown in green) that are involved in cell fate decisions for the RSCs' transition to the COP or I1

**FIGURE 6:**
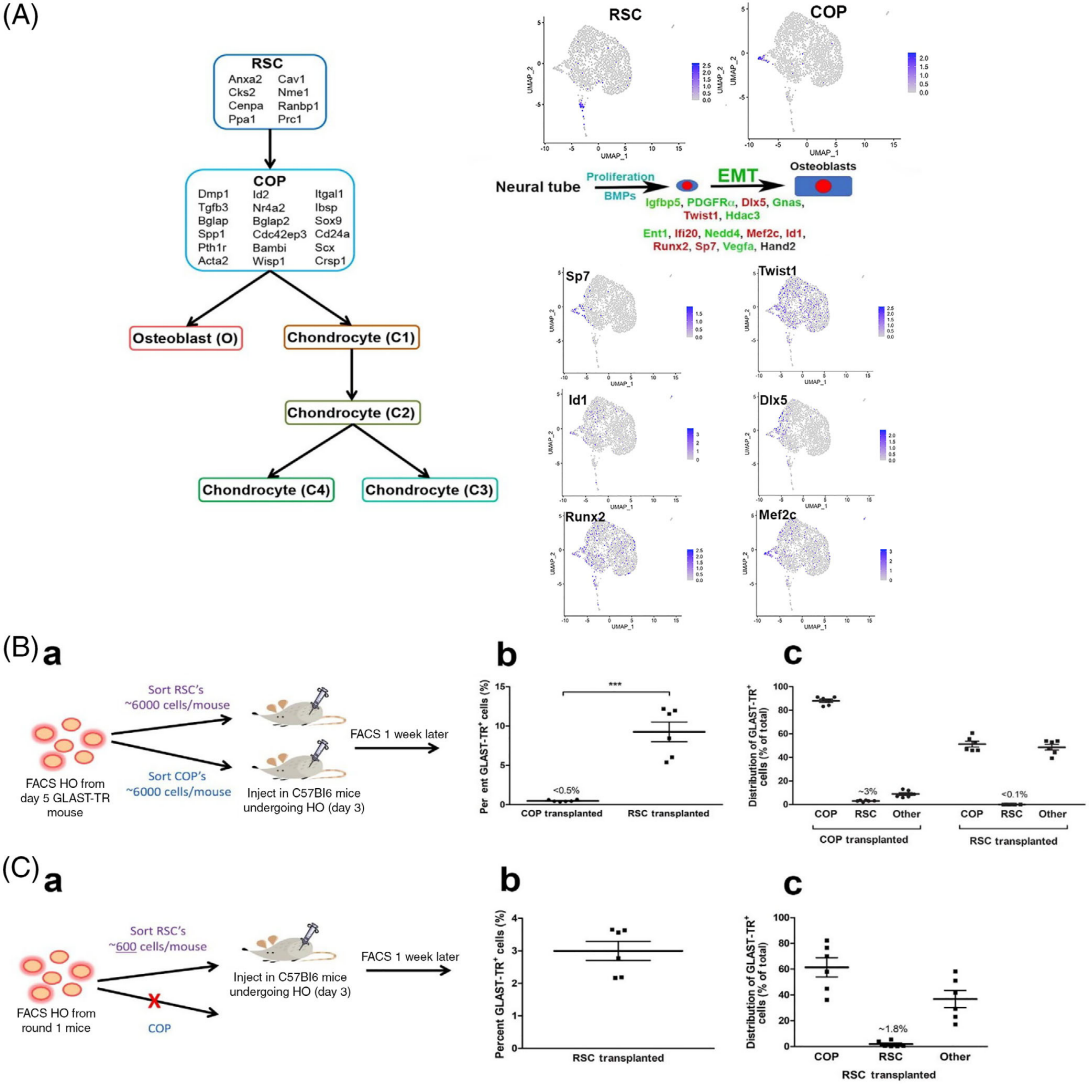
A, RSCs potentially undergo an epithelial to mesenchymal transition (EMT) to form COPs in BMP2‐induced bone formation. Dichotomy tree depicting the transition state genes derived from Figure 5 between RSCs and COPs. Neither I1 nor I2 are depicted in the dichotomy tree. The complete dichotomy tree shown was derived from the four BEAM heatmaps shown in Supporting Information Figure S1A by the procedure illustrated in Supporting Information Figure S1B. Additionally, a schematic of a tentative BMP2 EMT is presented in A. Genes listed were selected from the literature and their expression in the cells depicted through Seurat clusters (purple color denotes expression within the cell). Gene names are listed in red text if they appear to be specific to individual clusters and green text for those that are expressed but are found in multiple clusters. Only Hand2 transcript expression is totally absent (black). B, Serial transplantation confirms that RSCs function as stem/progenitors to repopulate COPs and form bone. B.a, BMP2‐induced HBF was established in C57BL/6 recipient mice (6/group) and 4 days later 3000 donor TR^+^‐RSCs or TR^+^‐COPs were injected into the two hind limbs of the mice undergoing HBF. COP (TR^+^Cd200^+^ Hmmr^−^) or RSC (TR^+^Hmmr^+^ Cd200^−^) donor cells were isolated by flow cytometry and injected into mice immediately after FACS isolation. Approximately 6000 cells were injected into the two hind limbs undergoing HBF in each recipient C57BL/6 mouse. Approximately 7 days later, GLAST‐TR^+^ cells were isolated from the hind limbs by collagenase digestion. The isolated cells were characterized by flow cytometry. B.b, Quantification of GLAST‐TR^+^ donor cells within the recipient HBF. Symbols represent data points, group averages (long horizontal bar) and SEM (short horizontal bars). One‐way ANOVA with Tukey correction showed statistically significant differences between the two groups shown in the bracket. ***P < .005. B.c, Characterization of the GLAST‐TR^+^ cells and quantification of COPs (TR^+^Cd200^+^ Hmmr^−^), RSCs (TR^+^Hmmr^+^ Cd200^−^) and other cells (TR^+^). Symbols represent data points, group averages (long horizontal bar) and SEM (short horizontal bars). C, Second transplantation of RSCs confirms their function as stem/progenitors and ability to repopulate COPs and form bone. C.a, Schematic depiction of the second transplantation experiment, now injecting approximately 600 cells. The red X depicts the termination of the transplantation experiments with COPs due to the limited number of cells that could be isolated from this first passage. C.b, Quantification of GLAST‐TR^+^ donor cells within HBF induced in recipient Bmp2‐induced C57BL/6 mice. C.c, Characterization of the GLAST‐TR^+^ cells and quantification of COPs (TR^+^Cd200^+^ Hmmr^−^), RSCs (TR^+^Hmmr^+^ Cd200^−^), and other cells (TR^+^)

To further characterize the transition between the RSC and COP, we used BEAM, a component of Monocle 2 that allows unambiguous determination of trajectory, to analyze and compare the transcriptomes of each of three clusters comprising a branchpoint, in this case branchpoint 1 (Figure 5) and provides a list of transcripts in each of these three cell types that are branchpoint dependent. Each branchpoint has an originating or transition cell type (Green color), which is the RSC in branchpoint 1 (Figure 5) and has the highest transcript values in the middle of the heatmap at pseudotime 0. The originating or transitioning branchpoint‐specific transcripts reach maxima somewhere in the center, nearer to the beginning of pseudotime. Analysis of branchpoint 1 revealed the RSC gives rise to both COP and I1 (Figure 5). This originating cell then differentiates into two other cell types, known as cell fate 1 (blue arrow, Figure 5), which is the COP for branchpoint 1 and has the highest transcript expression values at the right border and cell fate 2 (red arrow, I1 in branchpoint 1, Figure 5), where the highest values are at the left border. This analysis was performed for all four branchpoints (Supporting Information Figure S1A) and this analysis is then is used to generate the final trajectory. A diagram of this analysis procedure is shown in Supporting Information Figure S1B. The resultant trajectory was used to generate the dichotomy tree shown in Figure 6A.

Since the results of BEAM show the earliest cluster to be RSCs, we next analyzed the transition‐state transcripts elevated in the heat map (Figure 6) in the transition‐state for the RSC and COP clusters. These transcripts are depicted in the dichotomy tree boxes (Figure 6A). Surprisingly, these transcripts appear to be involved either in regulation of cell cycle (RSC) or associated with an EMT. To further determine whether the RSC could be undergoing an EMT to form an MSC (COP), we selected genes associated in the literature^36^, ^37^ with BMP2‐induced EMTs and then identified their cluster‐specific expression using Seurat mapping. Results of this targeted transcript analysis revealed that several of these transcripts are expressed in the RSC and/or COP clusters, but are absent from the other clusters (Figure 6A, red text). While only one of the transcripts (Figure 6A, black text) selected for analysis was absent from any transcriptome, several others were expressed in both clusters (Figure 6A, green text).

### 3.6 The RSC can engraft and form HFB through formation of the COP

To further confirm the stem/progenitor nature of these cells, both populations were isolated and characterized for their ability to undergo in vitro differentiation into osteoblast, chondrocytes, or adipocytes. Surprisingly, under basal culturing conditions RSCs rapidly replicated 50‐fold, whereas COPs expanded only 5‐fold during the same period. Cells were placed in differentiation media to generate mesenchymal lineages and 2 weeks later were analyzed for development of cartilage (Alcian blue), bone (Alizarin red), or adipocytes (oil red O) (Supporting Information Figure S2). RSCs were able to generate all three cell types when grown in specific medium (Supporting Information Figure S2) and matched results from our positive control (data not shown).

Alternatively, while COPs would undergo limited osteogenesis, only a handful of cells would undergo adipogenesis (Supporting Information Figure S3) when placed in specific media, suggesting these cells may be directed more toward osteoblasts than bone marrow‐derived mesenchymal stem cells. Problematically, COPs consistently died when placed in culture conditions to support chondrogenesis (Supporting Information Figure S2). However, since COPs did not replicate as extensively as RSCs, the pellet cultures consisted of one log fewer cells in each nodule, which may have contributed to their death.

To determine the stem/progenitor nature of RSCs and COPs, both TR^+^ populations were independently isolated and injected into recipient wild‐type mice (C57BL/6) undergoing HBF to determine their ability to engraft into BMP2‐induced HBF. Complete analysis of the transcripts produced during BMP2‐mediated HO in mouse shows that Hmmr is the only potential surface marker unique to RSCs. Therefore, TR^+^ RSCs were isolated by flow cytometry at day 4 after induction by selection for TR^+^ Hmmr^+^ Cd200^−^, likewise COPs were isolated at 10 days after induction by FACS for TR^+^ Hmmr^−^ Cd200^+^ cells (Supporting Information Figure S5). We injected 6000 cells per mouse (3000 cells per leg) into the hind limbs of C57BL/6 mice where HBF was induced 4 days prior. Four days (RSCs) or 10 days (COPs) later, cells were recovered from the muscle and analyzed by flow cytometry for the TR^+^ reporter as well as Hmmr and Cd200, to determine the engraftment of either labeled RSCs or COPs into BMP2‐induced heterotopic bone established in wild‐type mice. To maximize yields of these rare cells, RSCs were isolated much earlier than COPs because the trajectory in pseudotime (Figure 5D) shows that RSC are produced earlier than COPs.

Both RSCs and COPs were able to replicate in vivo and contribute to bone formation. In mice that received RSCs, approximately 9% of the total cells in HBF were found to be TR^+^ (Figure 6B.b). COPs engrafted at significantly lower levels, accounting for less than 0.5% of the total cells in HBF (Figure 6B.b). One possible reason for the lower engraftment of COPs into the tissues may be their lower rate of replication compared with RSCs. When 3000 RSCs were injected into the hind limb, approximately 138 951 ± 46 030 TR^+^ cells/limb were obtained 1 week later. Alternatively, after 1 week we obtained approximately 7385 ± 1909 TR^+^ cells/limb when we input 3000 COP cells. Thus, COPs replicated approximately 15‐25‐fold less than RSCs, supporting the reduced replication rate observed in the in vitro cultures.

Analysis of the types of cells within the GLAST‐TR^+^ donor population showed that most cells derived from the donor COPs were COPs (85%), whereas less than 5% were RSCs and 15% were other presumably more differentiated cells (Figure 6B.c). Alternatively, approximately 50% of the GLAST‐TR^+^ cells derived from the donor RSCs were found to express COP‐specific markers, 0.1% expressed RSC markers, and 49% were other presumably differentiated cells (Figure 6B.c). To further confirm the stem/progenitor nature of RSCs, we isolated GLAST‐TR^+^ Hmmr^+^ Cd200^−^ cells from these mice and reinjected them into wild‐type mice 4 days after induction with BMP2 and allowed them to engraft for 1 week into these mice undergoing BMP2‐induced HBF. In the second round of transplantation, 300 RSCs resulted in 44 984 ± 10 710 TR^+^ cells/limb. These results were like the first round of transplantation (Figure 6C.a,C.b), with a small proportion of the total cells isolated from the hind limb, possessing the donor tag. Furthermore, analysis of these donor‐derived cells showed again that most of the cells were either COPs or other cells and approximately 1% RSCs (Figure 6C.c).

The RSCs appear able to self‐renew, since starting at 3000 TR^+^ cells they expand to approximately 140,00 TR^+^ cells over the course of 1 week. During this same time period, they can also repopulate other cell types since starting with zero or almost zero TR^+^ COPs, TR^+^ RSCs expanded and then differentiated to approximately 70 000 COPs.

## 4 DISCUSSION

Here we used a GLAST‐TR mouse to identify and characterize a novel stem/progenitor cell (RSC) that is highly replicative. Robust in vitro and in vivo studies demonstrate that this unique cell population is capable of transitioning to a chondro‐osseous stem/progenitor cell (COP), which has a transcriptome like that of a mesenchymal stem cell, during BMP2‐induced HBF. Analysis of the RSC transcriptome suggests that these cells are epithelial in nature and they appear to undergo an EMT. Surprisingly, RSCs distribute a high proportion of their transcriptome to DNA replication and possess very few surface markers, suggesting a similarity to cancer stem cells.^38^ These cells continue to be highly replicating in vitro, and upon addition of specific differentiation media produce adipocytes, osteoblasts and chondrocytes. When isolated TR+ RSCs were injected into wild‐type mice undergoing HBF and allowed to engraft viable RSCs persisted and amplified to produce COPs. Engrafted RSCs readily formed COPs and mature cells, with only a few cells remaining as RSCs, suggesting that BMP2‐induced HBF favors their differentiation. Although only 9% of the cells were TR+ from the total population of cells isolated from the BMP2‐induced C57BL/6 mouse hind limb (Figure 6B.b); we have previously found that cells involved in HBF are the minority of cells within the hind limb. In studies using the GLAST‐TR+ mice, approximately the same level (7‐12%) of the mouse hind limb cells are positive for TR (Figure 1C). Alternatively, in vivo engraftment studies confirmed that COPs survived when transplanted and approximately 15% were other cell types, most (85%) stayed COPs and approximately 5% assumed an RSC phenotype. These data also support the branchpoint analysis, which suggests that RSCs are an early stem/progenitor cell and transition to a COP. In addition, we found that COPs are similar, but not identical, to mesenchymal stem cells. The mesenchymal nature of these cells was confirmed by analysis of the transcriptome, which showed that 2% of COP transcripts are involved in commitment to the mesenchymal lineage. Furthermore, bioinformatic analysis of the cell trajectory produced during HBF strongly suggests that COPs function as the mesenchymal intermediate for both osteoblasts and chondrocytes. Surprisingly, COPs expressed both early and late stage chondrocyte‐ and osteoblast‐specific transcripts, suggesting they hold the potential to further differentiate.

Recently, Debnath et al^7^ described a periosteal‐derived stem cell (PSC) that possesses a transcriptome similar to the COP. The PSC is described as a stem cell that mediates intramembranous bone formation and was identified through its expression of cathepsin K and Cd200. COPs not only express these transcripts, but also the transcripts for PDGFα, Cd90, Cd44, and Cd105, but these markers were not specific to this population and were expressed on some RSCs as well as more differentiated cell clusters. COPs also expressed GLAST but not GFAP, suggesting they are not like the cells characterized by Kan et al.^12^

However, COPs were unable to survive culturing when plated, with more than 50% cell death. Furthermore, the cells failed to replicate well, and while a few underwent osteogenesis, far fewer cells appeared to undergo adipogenesis. Also, when placed in pellet culture conditions COPs died instead of forming chondrocytes. However, the pellet culture methodology required very large numbers of cells, which were difficult to obtain by flow cytometry, leading to dependence on replication prior to establishing the pellets. Perhaps during this time the cells started to either differentiate or undergo senescence.

Analysis of cartilage and bone during HBF showed that most chondrocytes on day 6 and osteoblasts on day 14 were TR^+^. Surprisingly, there was a percentage of chondrocytes and osteoblasts that did not appear to express the reporter, suggesting the potential contribution of other mesenchymal stem/progenitors to this de novo bone formation. Flow cytometry analysis suggested that the GLAST‐TR^+^ cells peak in 2 weeks and then decline. Previous studies using this model showed the peak of bone formation at 2 weeks, before remodeling to a smaller persistent structure.^16^ By day 21, there is a significant decrease in the number of GLAST‐TR^+^ cells, which may be due in part to a decrease in cellularity of the bone itself. At this stage, the large chondrocyte population was no longer a substantial part of the tissue. Furthermore, this de novo bone was no longer growing, which suggests a decrease in stem/progenitor cells. We predict that the lack of ongoing growth would account for the decreased population; however, we cannot rule out that the initial unlabeled osteoblasts may persist to a greater extent than the GLAST‐TR^+^ cells contributing to this imbalance.

In tamoxifen pulsing studies, we found that cells were labeled with TR within 48 hours or early in the process, supporting the idea that GLAST expression is restricted to the RSCs and that they are the earliest cell type. While no other structures initially appeared labeled in the hind limb, the origin of RSCs is still unknown, and potentially an earlier GLAST‐negative stem/progenitor may also contribute. Alternatively, BMP2 may induce epigenetic reprogramming of adult cells in the mice that lead to RSCs. RSCs have a longer sorting time compared with the total population, due to their very low number and the necessity for multiple marker selection, thus exposing RSCs to a longer ex vivo period. Alternatively, we cannot rule out that RSC engraftment may be more efficacious when combined with COPs or another cell type within the total GLAST‐TR^+^population. There are several limitations to the studies described here, the first of which is the suggestion that the RSC resembles a stem‐like cell or cancer stem cell. The comparison to a stem‐like cell is because the RSC both self‐renews and differentiates to another cell in the lineage. Although we feel confident that the RSC is very early in the musculoskeletal lineage of HBF, we cannot assure that it is the most primitive cell of that lineage. Also, we have not yet shown that a single clone of the RSC will self‐renew and differentiate. The suggestion that the RSC is similar to a cancer stem cell was inferred from a comparison of the most abundant transcripts of the RSC to other transcripts found in the literature‐derived data‐mining database called Pathway Studio. When the top 225 transcripts from the RSC were entered into the database and a question asked of the relationship of the input transcripts to human disease, the top disease found was human cancer with 112 out of 225 RSC transcripts related to human cancer with a *P* value of 2.3 × 10^−28^. One of the related transcripts, Birc5 (survivin), had 104 references linking it to various human cancers. Other cancers (75 types) followed the overall listing of “human cancer” and one of these was glioblastoma (GBM), an uncurable brain cancer. Recently it has been proposed that BMP permits the cancer stem cell in GBM to remain quiescent and resist treatment.^39^ Also, it has been published that Hmmr is highly expressed in GBM tumors where it supports the self‐renewal and tumorigenic potential of the glioblastoma cancer stem cell.^40^ Therefore, although there is some data on the RSC being related to a cancer stem cell, it is only correlational and remains to be proven. Similarly, the suggestion that the RSC undergoes an EMT is supported by data that some of the transcripts in the branchpoint from the RSC to the COP either directly regulate factors known to be involved in EMT such as Snai1 and 2 and Twist1 and 2 or express transcripts such as Igfbp5 or Gnas that are involved directly in EMTs. However, this is also correlational data and perhaps suggests an EMT; however, this does not prove that one takes place. Another limitation of these studies is that although we have isolated and cultured the RSC from mouse, the similar isolation and culturing of such a cell from humans remains a formidable task. As noted above, we have not ruled out cooperation between stem/progenitor cells and either other stem/progenitor cells or other cells, such as macrophages, in the final trajectory of cell types involved in heterotopic ossification. Finally, we only tested RSCs for transition to mesenchymal lineages, but perhaps they can contribute to other tissues.

## CONCLUSION

The high level of replication with limited surface antigens indicates RSCs may be useful as an adult stem cell therapy. These cells hold the potential to avoid graft vs host responses even in the context of an allograft, while expanding locally to contribute to tissue regeneration. Thus, by use of these cells it is conceivable to either enhance musculoskeletal regeneration or to amplify bone formation to such an extent that it may take place where it ordinarily would not, such as during extensive bone injury or in the presence of bone disease.

## CONFLICT OF INTEREST

The authors declared no potential conflicts of interest.

## AUTHOR CONTRIBUTIONS

J.M.: collection and/or assembly of data; E.S.: collection and/or assembly of data, data analysis and interpretation, manuscript writing, conception and design; C.S.: collection and/or assembly of data, data analysis and interpretation; Z.G.: collection and/or assembly of data, data analysis and interpretation, manuscript writing; E.A.O.D.: conception and design, collection and/or assembly of data, manuscript writing; A.R.D.: bioinformatics data analysis, conception and design, manuscript writing, financial support, final approval of manuscript.

## Supporting information


**Table S1** Transcripts (Top 50) for each cluster indicated.

**Table S2** GO analysis to identify cluster cell types.

**Table S3** Expression of the Gt(Rosa)26Sor in all clusters.

**Table S4** Chondrocyte‐ and osteoblast‐associated transcripts expressed in individual Seurat clusters. New methods described in detail in Seurat version 3 were used to visualize the chondrocyte‐ and osteoblast‐specific transcripts in individual clusters. We have also shown Dot Plots of chondrocyte transcripts in clusters C1‐C4 as well as osteoblast transcripts in cluster O. References for some of the feature markers are as follows: Runx 2,^1^ Bglap,^2^ Spp1,^1^ Isbp,^1^ Ogn,^3^ Col1a2,^3^ Sp7,^4^ Sox9,^5^ Col6a1,^6^ Sparcl1,^7^ Prdx5,^8^ Aspn,^9^ Loxl2,^10^ Matn4,^11^ S100a11,^12^ and Fmod.^11^

**Figure S1A** BEAM all four branchpoints.

**Figure S1B** Process for conversion of BEAM to a dichotomy tree. In the diagram, each BEAM heatmap depicts one branchpoint. Each heatmap also has three sections, one section for the initiating cell type, one section for cell type 1 (rightward (blue arrow) and one section for cell type 2 (leftward, red arrow). The cell type for each section of the heatmap is determined by the predominant transcripts indicated to the right of that section of the heatmap. Zoom in to read those transcripts. Once these three cell types have been determined (initiating cell type and cell types 1 and 2), they can be placed into a triangle giving what has happened in that section of the dichotomy tree. After four triangles have been completed, one for each branchpoint, they can be locked together in one and only one trajectory to give the final trajectory. I2 is probably a contaminant and has been deleted from the final dichotomy tree shown in Figure 6A.

**Figure S2** Differentiation of RSCs and COPS into osteoblasts, chondrocytes, and adipocytes. RSCs and COPS cells were isolated and immediately placed in culture and allowed to replicate. After 2 weeks, RSCs were confluent, and the COPs did not appear to be further replicating. Cells were then switched to osteogenic, chondrogenic, or adipogenic differentiation media. After 2 weeks, cells were stained for tissue specific markers.

**Figure S3** Schematic depiction of Birc5 and Ki67 transcriptome expression in the RSC cluster using Seurat v.3.

**Figure S4** GLAST‐Cre^Ert2^:tdTomato red (TR)^floxSTOPflox^ mice induced with BMP2 on day 0 and euthanized on day 5 do not express the red reporter. Glast‐Cre^Ert2^:tdTR^floxSTOPflox^ mice (n = 4 per group) were induced with BMP2 on day 0 and then either treated with vehicle or tamoxifen daily for 5 days. Another group of mice was not induced with BMP2 on day 0 and then treated with tamoxifen daily for 5 days. A, Shows the percentage of TR^+^ cells made by each group. **P < .001; *P < .05. B.a, Analytical FACS of a BMP2^+^Tam^−^ mouse. B.b, Analytical FACS of a BMP2^+^Tam^+^ mouse.

**Figure S5** FACS isolation of the RSC and COP. Two groups of GLAST‐Cre^Ert2^:tdTR^floxSTOPflox^ mice (n = 8 per group) were injected with BMP2‐producing cells on day 0 and with tamoxifen each day until the mice were euthanized on day 7. The limb tissue was obtained and the cells from it prepared for sorting as described in the Materials and Methods. A, Each group of cells was reacted with antibodies against Hmmr and Cd200 followed by reaction with secondary antibodies containing BV421 (Hmmr) and Alexa fluor 488 (Cd200). This group was subjected to FACS and the COP isolated by taking the cells that were TR^+^Cd200^+^. B, The other groups of cells were subjected to FACS and the RSC isolated by taking the cells that were TR^+^Hmmr^+^. C, The COP isolation procedure was validated, and the purity of the cells checked by fixing the isolated cells (TR^+^Cd200^+^) for 15 minutes with 4% paraformaldehyde in PBS and subjecting them to analytical FACS. The profile shows almost complete purity of the cells since they are almost all Hmmr negative, as expected. D, The RSC isolation procedure was validated, and the purity of the cells checked by fixing the isolated cells (TR^+^Hmmr^+^) and subjecting them to analytical FACS. The profile shows almost complete purity of the cells since they are almost all Cd200 negative.

References for supplemental information.

## Data Availability

The complete RNA sequencing data contained in this manuscript are being made available by deposit to the NCBI GEO DataSets.
