# Supplementary material for: A replicating stem‐like cell that contributes to bone morphogenetic protein 2‐induced heterotopic bone formation
Source: Stem Cells Transl Med. 2020 Nov 27;10(4):623–35. doi: 10.1002/sctm.20-0378 (PMC7980206; doi:10.1002/sctm.20-0378)
Supplement: Supplementary file 1 — Table S1 Transcripts (Top 50) for each cluster indicated. [file SCT3-10-623-s007.pdf]

Supplemental Table 1:

Cluster C1		Cluster C2		Cluster C3		Cluster C4	
**Gene**	**p-value**	**Gene**	**p-value**	**Gene**	**p-value**	**Gene**	**p-value**
Matn4	2.741041e-158	Tnc	2.809192e-148	Clec3b	6.797004e-179	F3	4.886963e-42
Mgp	8.928914e-141	Lgals1	1.817392e-124	Smpd3	1.120946e-138	Igfbp7	9.009356e-34
Dpt	8.809896e-113	Tnn	2.945189e-124	Mgp	3.028737e-138	Hsd11b1	3.114747e-31
Gpx3	6.171720e-107	Serping1	3.595941e-112	Gsn	1.479452e-135	Sparcl1	6.023146e-30
Fmod	3.945531e-99	C1qtnf3	1.519302e-108	Col6a5	9.009924e-128	Serpina3n	1.335069e-27
Mfap4	1.313650e-91	Csrp1	1.015049e-99	Daglb	1.329182e-123	Ccl11	1.980115e-27
Aspn	7.607336e-88	Cthrc1	4.306804e-99	Gm9780	7.273830e-123	Mgp	6.039644e-27
Ly6a	1.450996e-87	Actg1	2.263431e-94	Ccl11	2.148189e-115	Cst3	2.000029e-26
Cpxm2	2.128201e-84	Lrrc15	2.881078e-90	Tnxb	7.181828e-115	Cygb	2.293684e-25
S100a6	2.256494e-83	Scx	1.130298e-86	S100a6	2.024751e-111	Serpina3g	3.492370e-24
Penk	1.199808e-82	Marcks	1.512997e-85	Bgn	7.963837e-103	Col6a2	1.241202e-23
Itgbl1	1.030746e-81	Col12a1	9.890234e-79	Cd248	2.275628e-100	Actg1	8.996800e-23
Ogn	1.098097e-80	Clec3b	2.660168e-77	Fn1	2.335886e-98	Vcam1	3.582200e-21
Ly6c1	3.614714e-77	Tgfbi	1.745393e-76	Adamts5	7.225325e-98	Fbln1	4.965137e-20
Tppp3	9.033356e-70	Lmna	2.413078e-76	Plpp3	1.894913e-92	Cthrc1	9.502595e-20
Col8a1	3.983323e-69	Acan	2.553856e-75	S100a16	1.178017e-89	S100a10	1.352595e-19
Pdgfrl	4.757974e-69	Angptl2	2.102298e-74	Anxa3	8.831485e-89	Bgn	9.171306e-19
Rps27	6.631058e-69	Rgs3	4.121184e-73	Dpt	1.541467e-88	Sepp1	1.011278e-18
Tnxb	1.264484e-67	Fth1	7.931759e-68	Ly6a	7.976950e-87	Rgs16	1.281056e-18
Clec3b	2.009152e-66	Tpm4	2.978927e-67	C3	5.212278e-86	Col6a1	3.890834e-18
Comp	6.603346e-64	Tpm2	1.004918e-66	Marcks	2.408820e-85	Il33	4.380916e-18
Cst3	7.412173e-59	Gsn	6.688558e-66	Tnfaip6	7.823682e-80	Ptn	1.616561e-17
Klf2	8.327884e-58	Serpinh1	7.898342e-66	S100a13	2.104048e-79	Apoe	1.583153e-16
Cd248	1.255636e-57	Acta2	2.456117e-65	Aspn	9.713033e-79	Smoc2	4.120776e-16
Pfn1	1.556296e-57	Inhba	2.857475e-64	Gas7	3.622493e-77	Rbp1	4.590173e-16
Itm2a	1.154241e-56	Aspn	5.482852e-64	Ly6c1	1.067410e-76	Mylk	1.260342e-15
Clec11a	1.632163e-56	Tm4sf1	3.614860e-62	Olfml3	1.295732e-75	Enpp2	3.158888e-15
S100a10	8.284399e-55	Vim	5.185881e-62	Cd34	3.495992e-75	Tppp3	5.506106e-15
Gsn	9.289033e-55	Sepp1	4.680114e-61	Rarres2	7.850049e-75	Tmem176b	3.946535e-14
Smoc2	1.047827e-52	Wisp1	2.599907e-59	Car4	9.199746e-74	Anxa2	7.634538e-14
Rps27rt	9.890849e-52	Eno1	7.045936e-59	Ptx3	4.759261e-71	Ccl8	8.406188e-14
Cd34	1.011852e-51	Pkm	1.424704e-58	Col5a3	7.577113e-69	Serping1	2.655687e-13
Anxa2	1.175168e-51	Enpp1	1.657598e-58	Dpep1	2.626466e-67	Mfap4	1.401346e-12
Bgn	3.157810e-50	Rarres2	1.396472e-56	Pi16	3.702905e-67	C1qtnf3	1.654015e-12
Nov	3.528458e-50	Itm2b	1.705745e-56	Prss23	6.616158e-67	Mest	2.108879e-12
Txn1	4.141510e-50	Prss23	2.729454e-56	Mfap4	2.116818e-64	Ctsl	2.218301e-12
C3	9.756288e-50	Myl12a	3.512418e-56	Fbn1	4.285449e-64	Tmsb10	4.853608e-12
Rpl18a	1.176689e-47	Csrp2	4.157419e-56	Basp1	5.991357e-64	Cd63	8.789881e-12
Atf3	2.416434e-46	Tpm1	4.775759e-55	Nid1	1.710678e-61	Loxl2	9.990953e-12
Tnfaip6	3.411750e-45	Myl9	4.205559e-54	Fmod	1.901190e-58	Bace2	1.504454e-11
S100a16	3.532515e-44	Tagln	2.546590e-53	Col6a6	2.376608e-58	Wisp2	1.703413e-11
Col6a2	4.096107e-44	Myl6	7.600233e-53	Ugdh	2.378112e-56	Gnas	1.822217e-11
Crip1	5.126065e-44	Plpp3	7.279920e-52	Pcolce2	2.982721e-56	B2m	2.746940e-11
Ifitm3	6.426698e-44	Ptma	8.854545e-52	Col8a1	4.069723e-56	Angptl4	2.999562e-11
Gm9780	2.582313e-43	Sfrp2	7.902635e-51	Clec11a	3.738575e-55	Pdgfrl	3.221198e-11
Malat1	4.449643e-43	Col14a1	8.513077e-49	Pdgfr1	2.999771e-53	Npc2	5.603493e-11
Col8a2	5.229009e-43	Sec61b	8.855329e-49	Cmah	4.594642e-53	Prss23	6.617379e-11
Maged2	1.239015e-42	Ppia	1.453580e-48	Heg1	5.895913e-53	Itm2a	1.079038e-10
Ccl11	2.39E-42	Arl4c	3.33E-48	Fstl1	4.19E-52	Pamr1	1.10E-10
Ptx3	2.44E-42	Ly6a	4.06E-48	Itih5	5.22E-52	Angptl1	1.65E-10

Cluster O		Cluster COP		Cluster RSC	
**Gene**	**p-value**	**Gene**	**p-value**	**Gene**	**p-value**
Gnas	1.028971e-114	Panx3	1.093052e-275	Birc5	5.610544e-226
Ly6a	3.725776e-113	Col11a2	8.052977e-185	Tpx2	1.883251e-200
Sfrp4	7.826493e-96	Slc13a5	1.777758e-171	Ccnb2	3.841857e-178
Igf1	4.349841e-91	Slc8a3	1.777758e-171	Hmmr	1.920278e-171
Gas1	4.192218e-89	Frzb	3.970200e-165	Cdkn3	1.464489e-166
Serping1	1.725129e-88	Hapln1	1.238036e-153	Ccna2	7.425084e-165
Ly6c1	1.050151e-84	Irx5	4.339095e-147	Cdc20	1.136755e-164
Sfrp1	8.719844e-79	Fzd9	1.460437e-142	Cenpa	2.124252e-148
Clec3b	1.014549e-72	Susd5	1.025090e-134	Fam64a	1.052031e-147
Dcn	1.752901e-72	Cox4i2	2.750947e-131	Ckap2	3.755608e-144
Igfbp4	9.640843e-70	Cspg4	7.061627e-130	Kif20a	7.728137e-136
Dpt	5.401736e-69	Ihh	2.829846e-124	Ccnb1	7.586899e-131
Fmod	2.367220e-66	Sp7	1.085803e-123	Cdca3	4.811473e-130
Ifitm3	3.470844e-60	Scube1	1.454399e-120	Cdca8	3.337857e-129
C4b	1.922857e-57	Col9a1	1.099460e-117	Knstrn	1.958745e-126
Tnxb	1.147836e-54	Col13a1	1.189687e-117	Racgap1	1.556244e-114
Sfrp2	2.758243e-54	Rab11fip4	1.114222e-114	Tk1	2.725789e-112
Mgst1	2.962985e-53	Cpe	2.409772e-113	Kif23	2.685165e-105
Plpp3	1.228389e-50	Fgfr3	2.428626e-110	Cenpm	1.280011e-102
Fbln2	2.920432e-50	Wif1	2.576588e-107	Cenpf	2.522895e-101
Fth1	4.443873e-50	Tcf7	6.183884e-105	Mki67	2.052780e-90
Mt2	9.603125e-49	Ifitm5	1.279178e-102	Top2a	4.400499e-88
Tnfaip2	1.533412e-46	Col9a3	6.822669e-90	Prc1	5.075844e-81
Has1	4.557045e-46	Sox6	4.262239e-89	Cks2	9.772164e-80
Plac8	7.274163e-46	Sdk2	1.053444e-86	Stmn1	1.419248e-76
Cfb	3.558594e-45	Nt5e	3.795062e-83	Lockd	1.057512e-71
Gas6	7.818921e-45	Bambi	3.541752e-82	Smc2	6.692888e-60
Itm2b	6.405101e-44	Zfp652	6.596287e-82	Spc25	1.142090e-58
Emb	2.004747e-43	Rcor2	9.191883e-82	Spc24	1.039645e-55
Cd248	7.581463e-42	Cgref1	2.065614e-80	Ube2c	3.779619e-55
Nfkbia	8.980433e-41	Mef2c	5.639818e-77	Tacc3	3.072143e-51
Angptl1	2.170879e-40	Alpl	4.123788e-76	Cenpw	1.610334e-46
Matn4	4.300426e-40	Ramp1	3.076657e-75	Lmnb1	2.506608e-45
Ltbp4	1.159672e-37	Smim5	4.732201e-74	Gmnn	4.003546e-45
Fbln7	2.831020e-37	Eps8l2	1.341510e-73	Rrm2	5.023286e-44
Col11a1	3.969448e-37	Cdo1	1.509906e-71	Rrm1	1.427268e-43
Ace	8.696552e-37	Rarres1	2.589203e-71	Hmgb2	2.339666e-42
Lbp	9.079148e-37	Slc16a4	1.151726e-68	Hmgb3	3.288601e-39
Cygb	7.226265e-36	Trpv4	1.892563e-68	Kif22	7.314794e-35
Mt1	1.968128e-35	Pth1r	2.636892e-66	Cdk1	1.193077e-33
Jund	3.336392e-35	Smpd3	2.885416e-61	H2afz	3.344592e-29
Il6	1.597075e-34	P3h2	6.052410e-60	Dynlt1a	1.331153e-27
Wisp2	2.448915e-34	Col9a2	5.083304e-58	Hmgn2	6.833672e-27
Apod	3.754725e-34	Sgms2	7.411236e-58	Smc4	1.202292e-25
Col12a1	8.240678e-34	Cd200	8.185589e-57	Rangap1	2.718394e-25
Ecm1	6.622881e-33	Chad	1.762056e-55	Tyms	7.932999e-25
Cd34	1.542556e-32	Unc5b	3.512273e-55	Nucks1	8.320930e-24
Entpd2	2.619010e-32	Kcnk1	1.15E-53	Hmgb1	2.089907e-23
Col14a1	3.088306e-32	C130050O18Rik	2.06E-52	Ran	8.39E-23
Mmp3	4.346630e-32	Ibsp	2.19E-51	Anp32e	6.31E-22
