# Supplementary material for: A replicating stem‐like cell that contributes to bone morphogenetic protein 2‐induced heterotopic bone formation
Source: Stem Cells Transl Med. 2020 Nov 27;10(4):623–35. doi: 10.1002/sctm.20-0378 (PMC7980206; doi:10.1002/sctm.20-0378)
Supplement: Supplementary file 2 — Table S2 GO analysis to identify cluster cell types. [file SCT3-10-623-s011.pdf]

Supplemental Table 2*:

Cluster	GO ID	GO label	pValue	Unique features of transcriptome
**Osteoblast (O)**	GO:0045667, GO:0045669 GO:0045778	Bone Morphogenesis, Positive regulation of osteoblast differentiation, Positive regulation of ossification	4.0×10^{-4} 9.3×10^{-7} 8.1×10^{-6}	
**Chondrocyte (C1)**	GO:0002063 GO:0002062 GO:0051216	Chondrocyte development, Chondrocyte differentiation, Cartilage development	1.4×10^{-10} 2×10^{-8} 4.6×10^{-8}	
**Chondrocyte (C2)**	GO:0002062 GO:0051216 GO:0003413	Chondrocyte differentiation, Cartilage development, Chondrocyte differentiation involved in endochondral bone morphogenesis	3.9×10^{-8} 2.9×10^{-6} 5.2×10^{-5}	
**Chondrocyte (C3)**	GO:0002062 GO:0061181 GO:1903041	Chondrocyte differentiation, Regulation of chondrocyte development, Regulation of chondrocyte hypertrophy	2.5×10^{-5} 8×10^{-2} 1.0	
**Chondrocyte (C4)**	GO:0051216 GO:0002063 GO:0003415	Cartilage development, Chondrocyte development, Chondrocyte hypertrophy	6.5×10^{-5} 4.5×10^{-3} 1.0	
**COP**	Indeterminant	Indeterminant		Expression of both osteoblast and chondrocyte transcripts
**RSC**	Indeterminant	Indeterminant		Very high percentage of transcripts involved in replication
**I1**	Indeterminant	Indeterminant		

*The top 100 transcripts determined for each cluster using Seurat (v3) were entered into the "Gene Enrichment Analysis" at geneontology.org and the results of the "cellular component" for mouse are reported.
