# Supplementary material for: A replicating stem‐like cell that contributes to bone morphogenetic protein 2‐induced heterotopic bone formation
Source: Stem Cells Transl Med. 2020 Nov 27;10(4):623–35. doi: 10.1002/sctm.20-0378 (PMC7980206; doi:10.1002/sctm.20-0378)
Supplement: Supplementary file 3 — Table S3 Expression of the Gt(Rosa)26Sor in all clusters. [file SCT3-10-623-s008.pdf]

Supplemental Table 3. Expression of the Gt(Rosa)26Sor[a]

Cluster	O	C1	C2	C3	C4	COP	RSC	I1
**Gt(Rosa)26Sor**	16	8.7	7.8	8.2	6.7	11.5	7.6	10.8

[a]The value of the transcript of Gt(Rosa)26Sor was determined in each cluster.
