# Supplementary figures and images for: A replicating stem‐like cell that contributes to bone morphogenetic protein 2‐induced heterotopic bone formation
Source: Stem Cells Transl Med. 2020 Nov 27;10(4):623–35. doi: 10.1002/sctm.20-0378 (PMC7980206; doi:10.1002/sctm.20-0378)

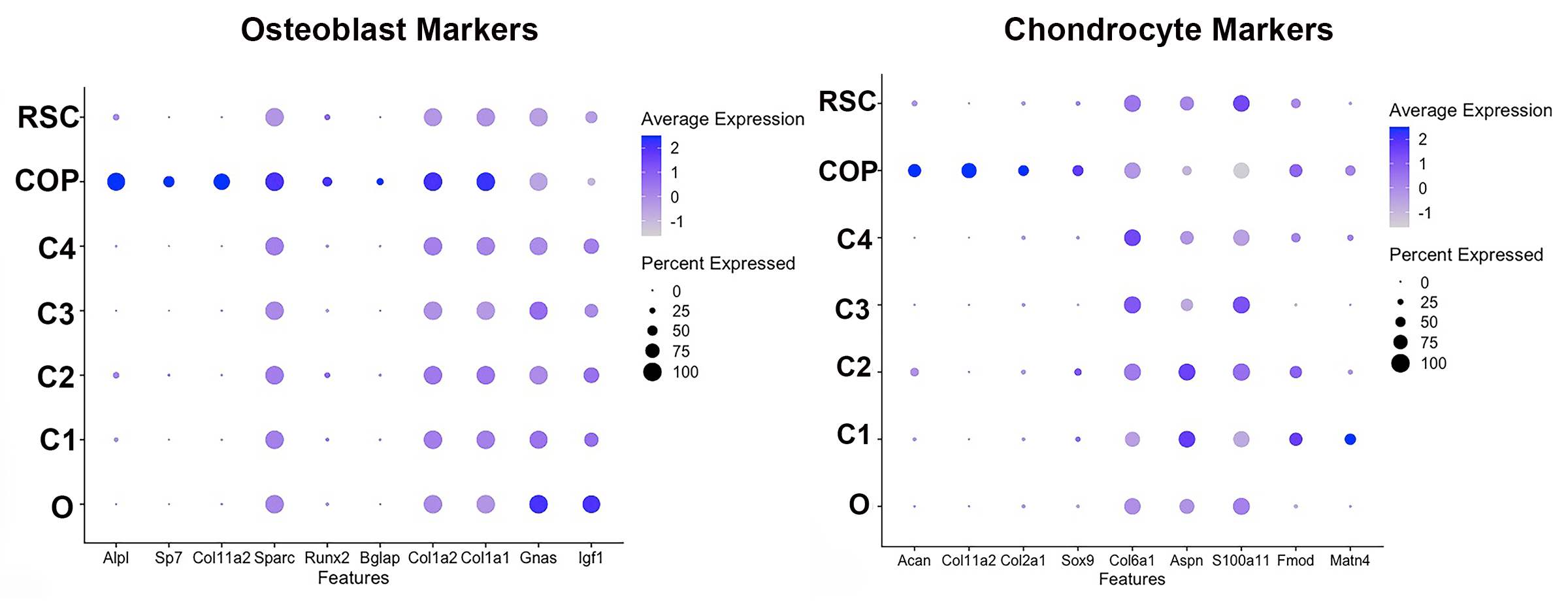

Supplement: Supplementary file 4 — Table S4 Chondrocyte‐ and osteoblast‐associated transcripts expressed in individual Seurat clusters. New methods described in detail in Seurat version 3 were used to visualize the chondrocyte‐ and osteoblast‐specific transcripts in individual clusters. We have also shown Dot Plots of chondrocyte transcripts in clusters C1‐C4 as well as osteoblast transcripts in cluster O. References for some of the feature markers are as follows: Runx 2,1 Bglap,2 Spp1,1 Isbp,1 Ogn,3 Col1a2,3 Sp7,4 Sox9,5 Col6a1,6 Sparcl1,7 Prdx5,8 Aspn,9 Loxl2,10 Matn4,11 S100a11,12 and Fmod.11 [file SCT3-10-623-s002.tif]

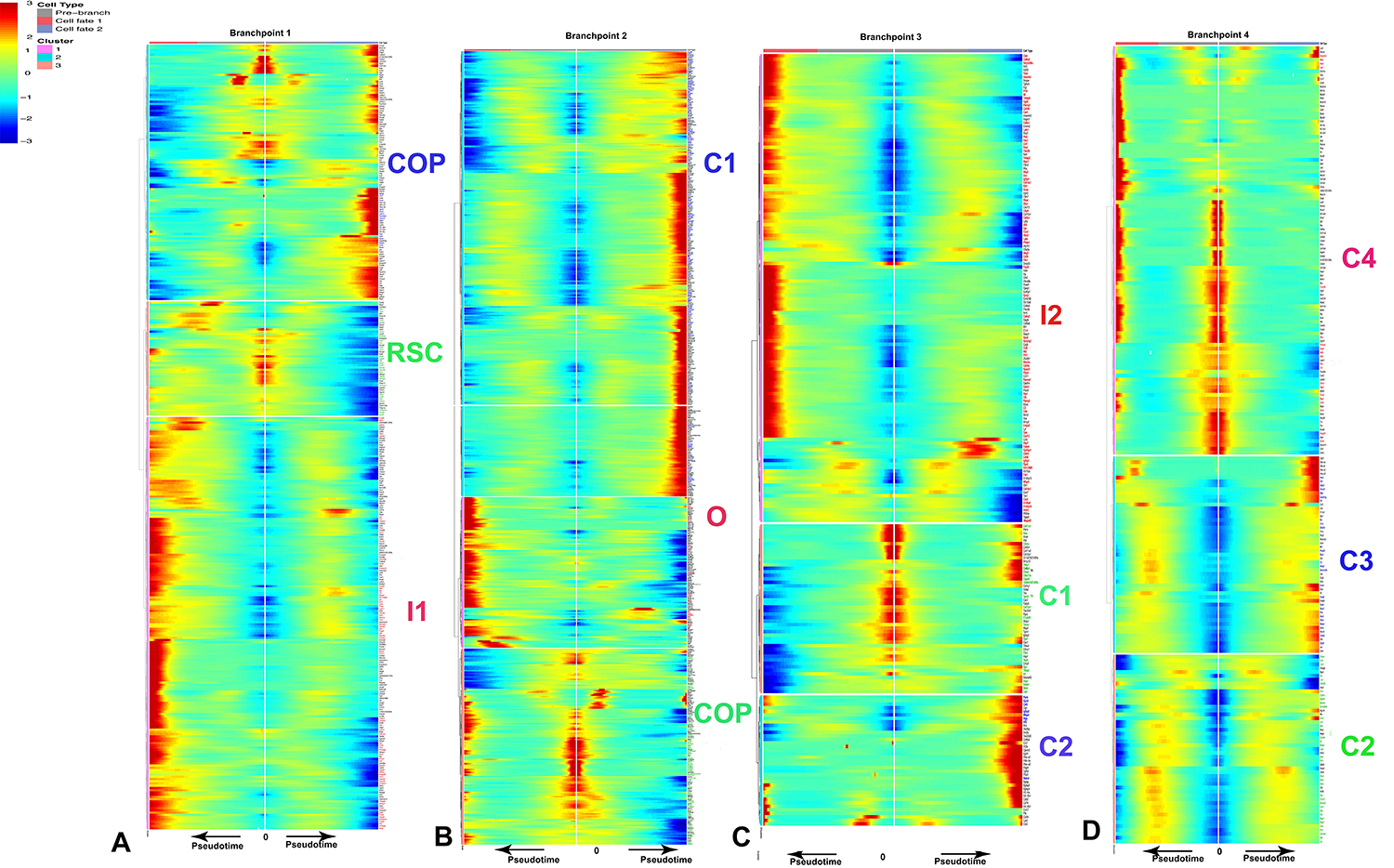

Supplement: Supplementary file 5 — Figure S1A BEAM all four branchpoints. [file SCT3-10-623-s010.tif]

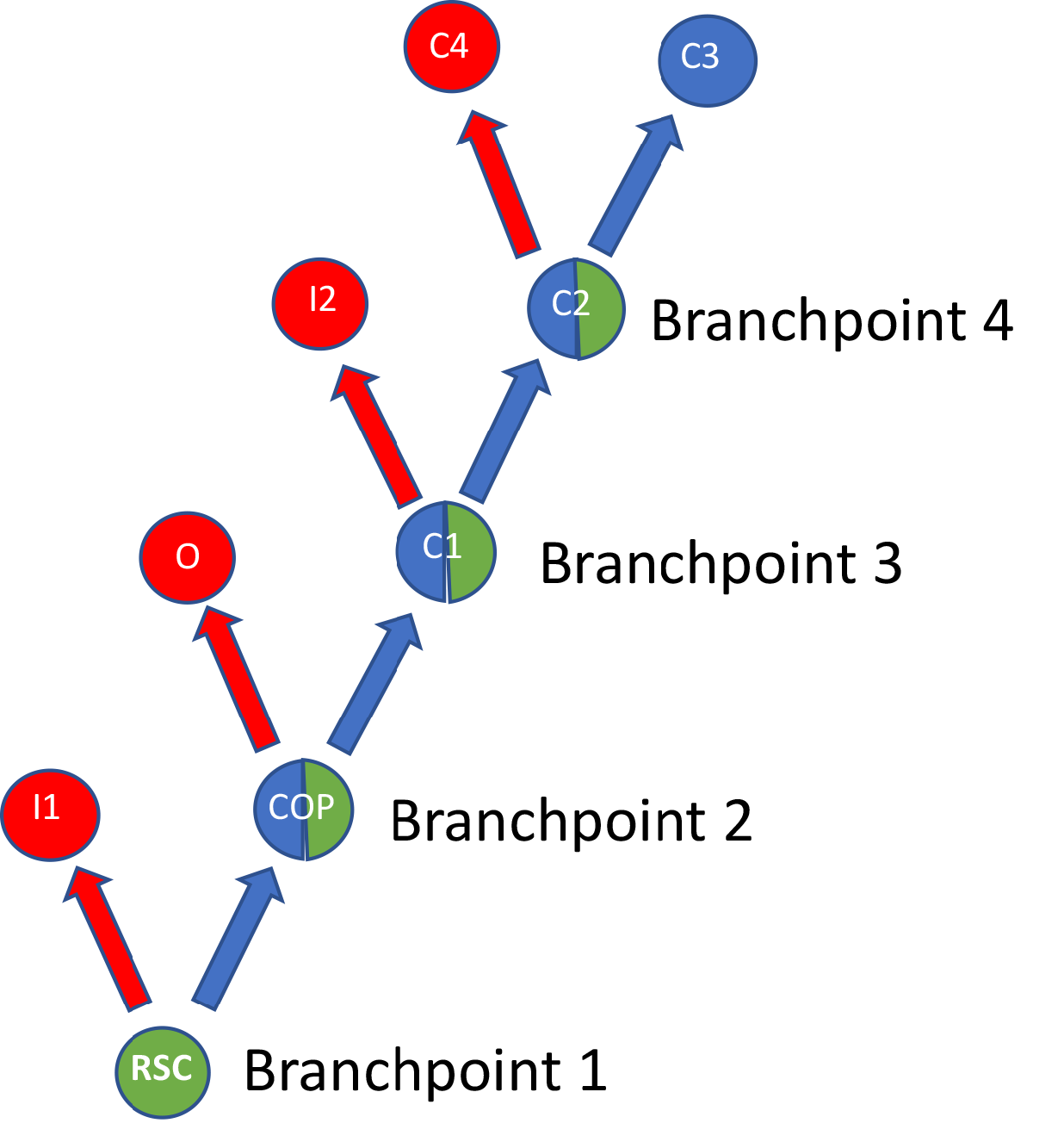

Supplement: Supplementary file 6 — Figure S1B Process for conversion of BEAM to a dichotomy tree. In the diagram, each BEAM heatmap depicts one branchpoint. Each heatmap also has three sections, one section for the initiating cell type, one section for cell type 1 (rightward (blue arrow) and one section for cell type 2 (leftward, red arrow). The cell type for each section of the heatmap is determined by the predominant transcripts indicated to the right of that section of the heatmap. Zoom in to read those transcripts. Once these three cell types have been determined (initiating cell type and cell types 1 and 2), they can be placed into a triangle giving what has happened in that section of the dichotomy tree. After four triangles have been completed, one for each branchpoint, they can be locked together in one and only one trajectory to give the final trajectory. I2 is probably a contaminant and has been deleted from the final dichotomy tree shown in Figure 6A. [file SCT3-10-623-s001.tif]

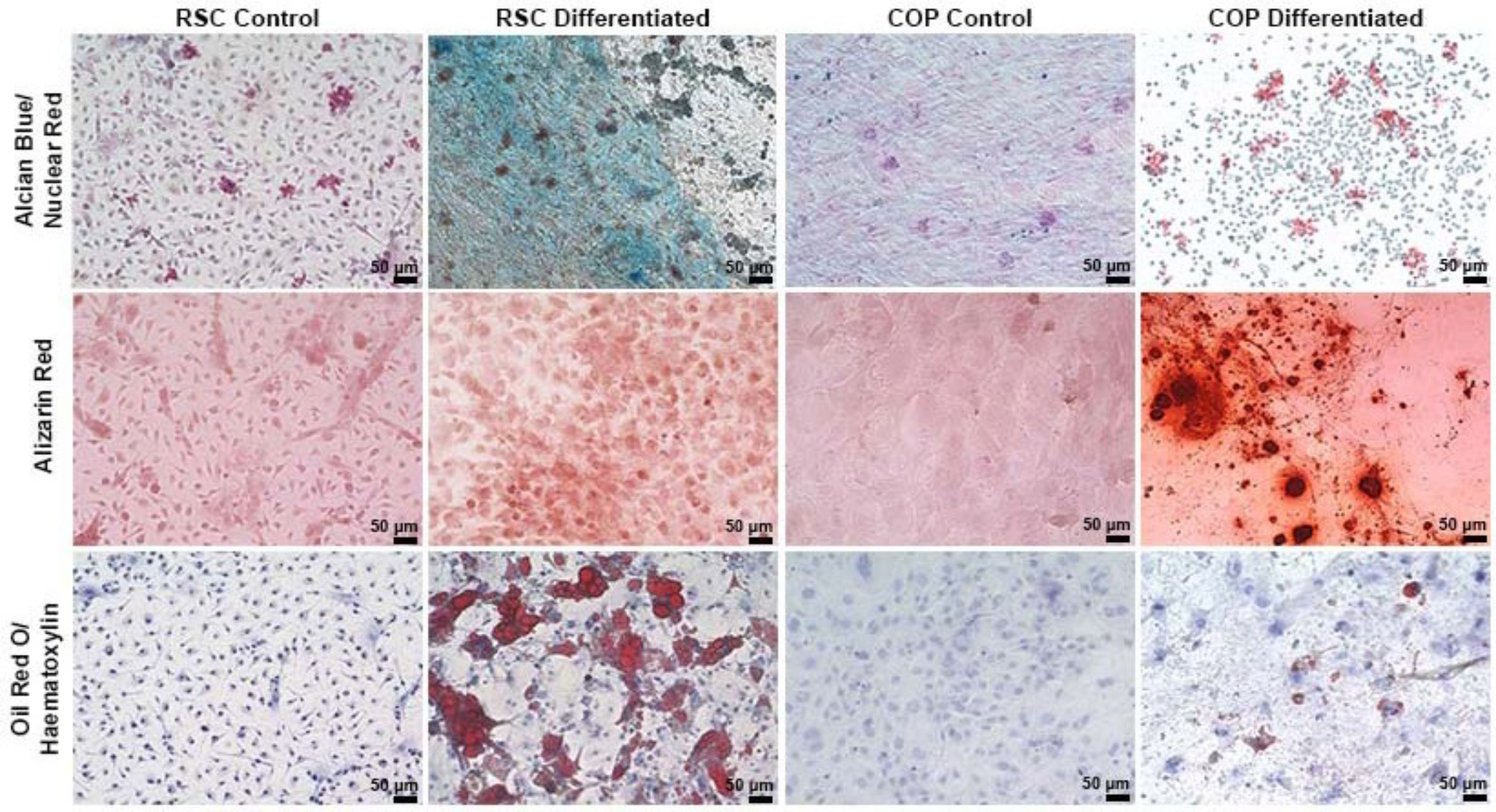

Supplement: Supplementary file 7 — Figure S2 Differentiation of RSCs and COPS into osteoblasts, chondrocytes, and adipocytes. RSCs and COPS cells were isolated and immediately placed in culture and allowed to replicate. After 2 weeks, RSCs were confluent, and the COPs did not appear to be further replicating. Cells were then switched to osteogenic, chondrogenic, or adipogenic differentiation media. After 2 weeks, cells were stained for tissue specific markers. [file SCT3-10-623-s006.tif]

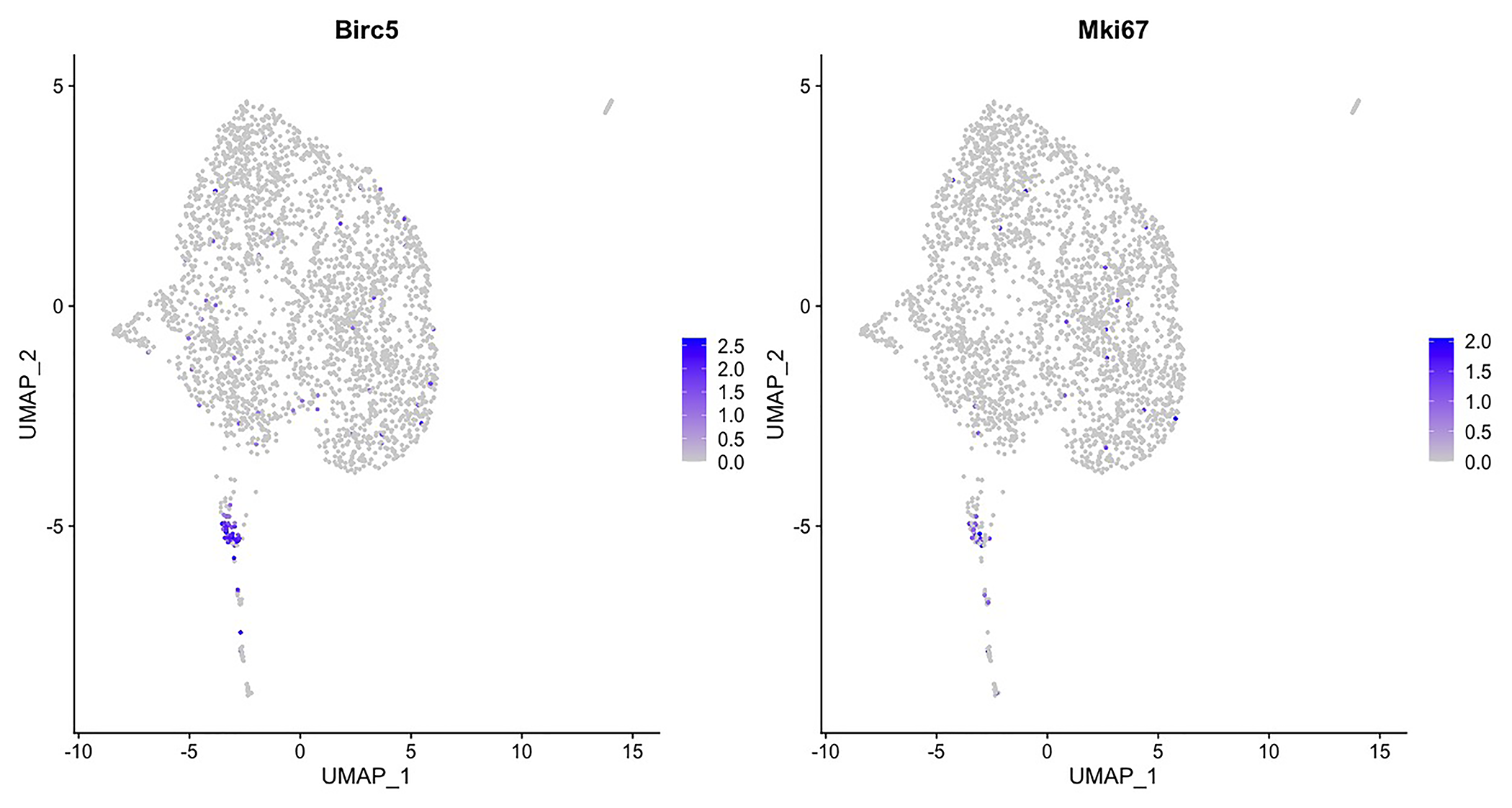

Supplement: Supplementary file 8 — Figure S3 Schematic depiction of Birc5 and Ki67 transcriptome expression in the RSC cluster using Seurat v.3. [file SCT3-10-623-s004.tif]

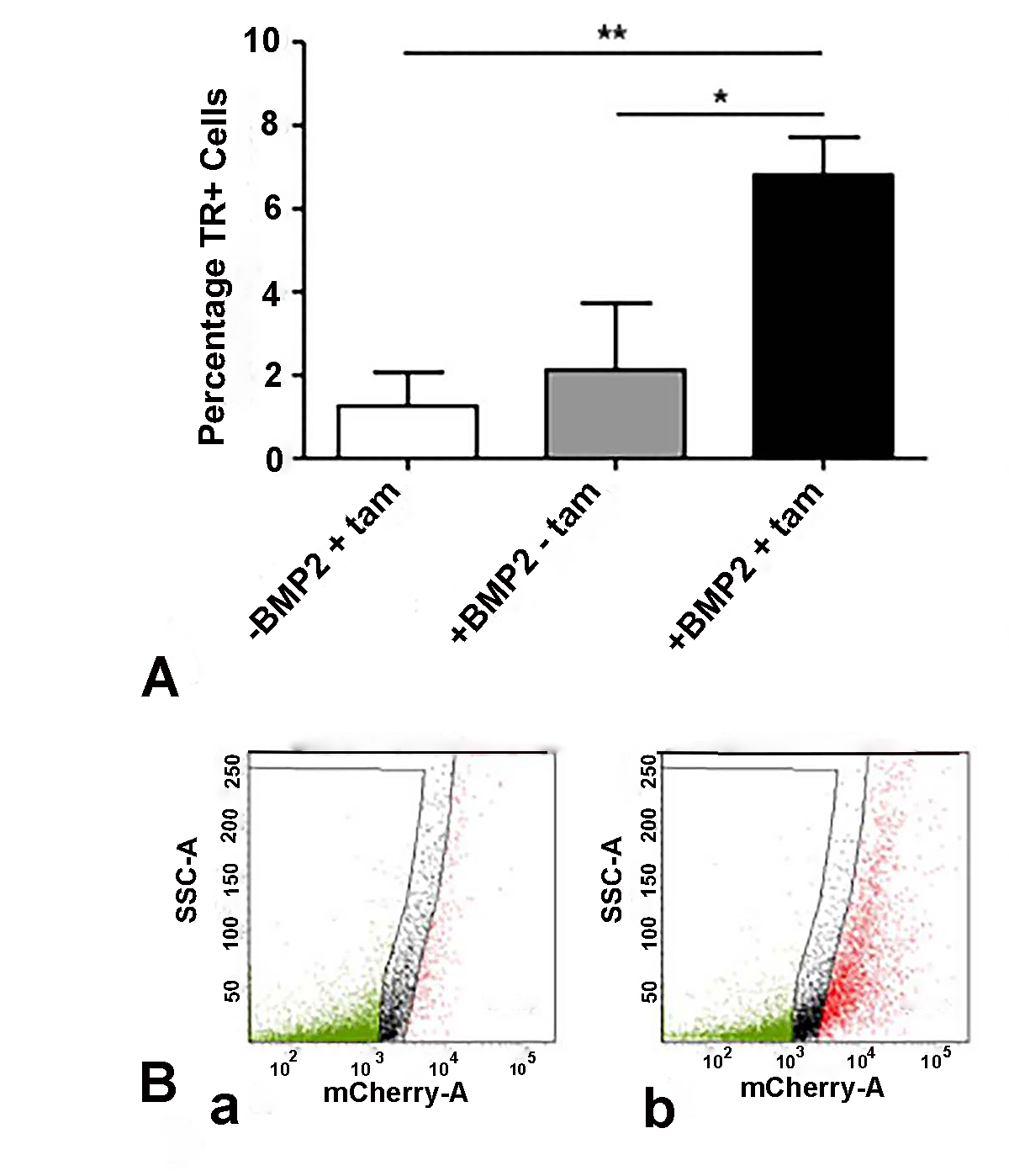

Supplement: Supplementary file 9 — Figure S4 GLAST‐CreErt2:tdTomato red (TR)floxSTOPflox mice induced with BMP2 on day 0 and euthanized on day 5 do not express the red reporter. Glast‐CreErt2:tdTRfloxSTOPflox mice (n = 4 per group) were induced with BMP2 on day 0 and then either treated with vehicle or tamoxifen daily for 5 days. Another group of mice was not induced with BMP2 on day 0 and then treated with tamoxifen daily for 5 days. A, Shows the percentage of TR+ cells made by each group. **P < .001; *P < .05. B.a, Analytical FACS of a BMP2+Tam− mouse. B.b, Analytical FACS of a BMP2+Tam+ mouse. [file SCT3-10-623-s003.tif]

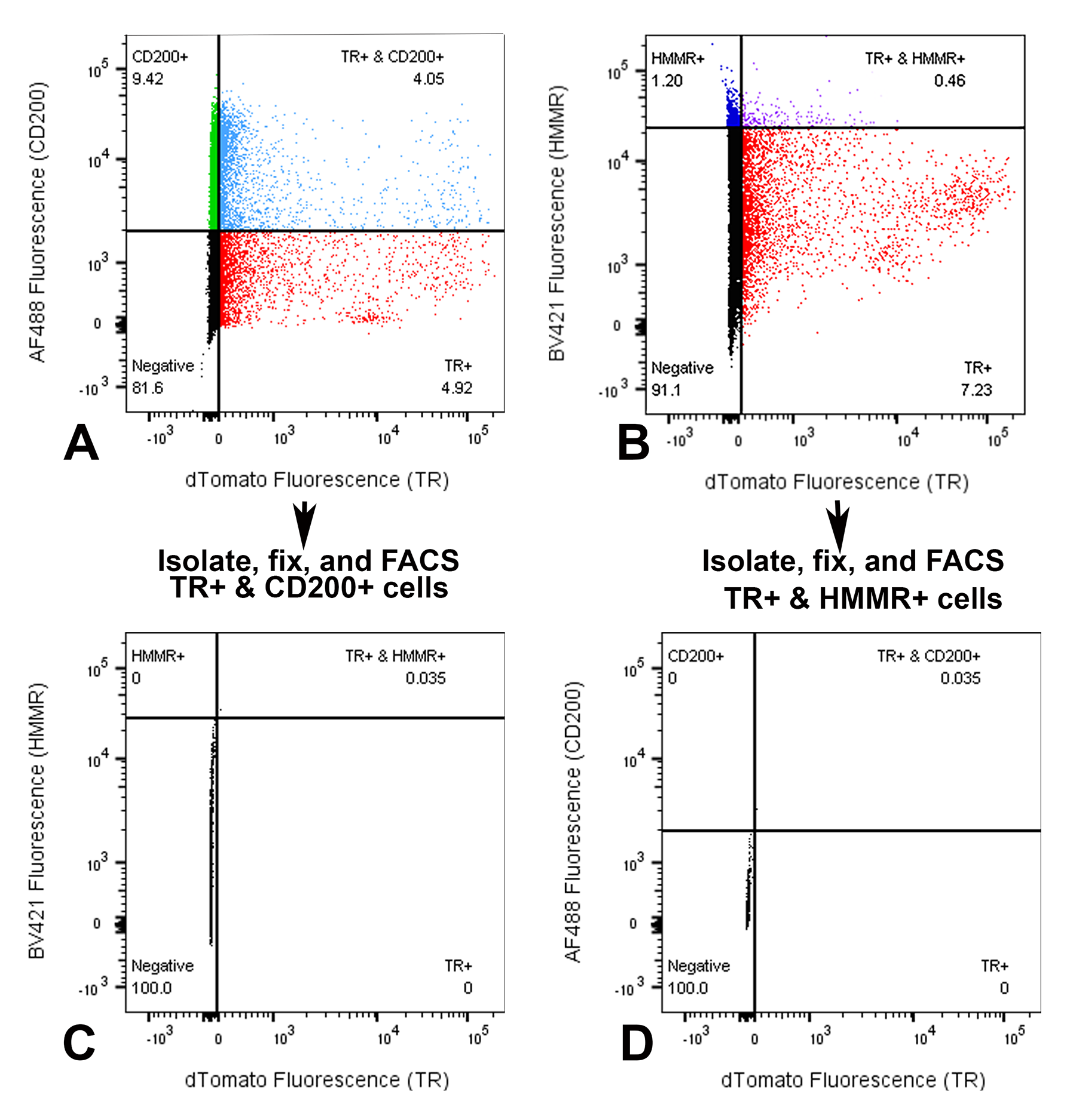

Supplement: Supplementary file 10 — Figure S5 FACS isolation of the RSC and COP. Two groups of GLAST‐CreErt2:tdTRfloxSTOPflox mice (n = 8 per group) were injected with BMP2‐producing cells on day 0 and with tamoxifen each day until the mice were euthanized on day 7. The limb tissue was obtained and the cells from it prepared for sorting as described in the Materials and Methods. A, Each group of cells was reacted with antibodies against Hmmr and Cd200 followed by reaction with secondary antibodies containing BV421 (Hmmr) and Alexa fluor 488 (Cd200). This group was subjected to FACS and the COP isolated by taking the cells that were TR+Cd200+. B, The other groups of cells were subjected to FACS and the RSC isolated by taking the cells that were TR+Hmmr+. C, The COP isolation procedure was validated, and the purity of the cells checked by fixing the isolated cells (TR+Cd200+) for 15 minutes with 4% paraformaldehyde in PBS and subjecting them to analytical FACS. The profile shows almost complete purity of the cells since they are almost all Hmmr negative, as expected. D, The RSC isolation procedure was validated, and the purity of the cells checked by fixing the isolated cells (TR+Hmmr+) and subjecting them to analytical FACS. The profile shows almost complete purity of the cells since they are almost all Cd200 negative. [file SCT3-10-623-s005.tif]
