# Supplementary material for: A replicating stem‐like cell that contributes to bone morphogenetic protein 2‐induced heterotopic bone formation
Source: Stem Cells Transl Med. 2020 Nov 27;10(4):623–35. doi: 10.1002/sctm.20-0378 (PMC7980206; doi:10.1002/sctm.20-0378)
Supplement: Supplementary file 11 — References for supplemental information. [file SCT3-10-623-s009.docx]

**REFERENCES FOR SUPPLEMENTAL INFORMATION**

1. Komori T. Signaling networks in RUNX2-dependent bone development. **J. CELL BIOCHEM.** 2011;112(3):750–755.

2. Marulanda J, Eimar H, McKee MD, et al. Matrix Gla protein deficiency impairs nasal septum growth, causing midface hypoplasia. **J. BIOL. CHEM.** 2017;292(27):11400–11412.

3. Pope FM, Nicholls AC, McPheat J, et al. Collagen genes and proteins in osteogenesis imperfecta. **J. MED. GENET.** 1985;22(6):466–478.

4. Baek W-Y, Lee M-A, Jung JW, et al. Positive regulation of adult bone formation by osteoblast-specific transcription factor osterix. **J. BONE MINER. RES.** 2009;24(6):1055–1065.

5. Huang W, Chung UI, Kronenberg HM, et al. The chondrogenic transcription factor Sox9 is a target of signaling by the parathyroid hormone-related peptide in the growth plate of endochondral bones. **PROC. NATL. ACAD. SCI. USA** 2001;98(1):160–165.

6. Hsueh M-F, Khabut A, Kjellström S, et al. Elucidating the molecular composition of cartilage by proteomics. **J. PROTEOME RES.** 2016;15(2):374–388.

7. Aeschlimann D, Wetterwald A, Fleisch H, et al. Expression of tissue transglutaminase in skeletal tissues correlates with events of terminal differentiation of chondrocytes. **J. CELL BIOL.** 1993;120(6):1461–1470.

8. De la Fuente A, Mateos J, Lesende-Rodríguez I, et al. Proteome analysis during chondrocyte differentiation in a new chondrogenesis model using human umbilical cord stroma mesenchymal stem cells. **MOL. CELL PROTEOMICS** 2012;11(2):M111.010496.

9. Duval E, Bigot N, Hervieu M, et al. Asporin expression is highly regulated in human chondrocytes. **MOL MED** 2011;17(7–8):816–823.

10. Iftikhar M, Hurtado P, Bais MV, et al. Lysyl oxidase-like-2 (LOXL2) is a major isoform in chondrocytes and is critically required for differentiation. **J. BIOL. CHEM.** 2011;286(2):909–918.

11. Wilson R, Norris EL, Brachvogel B, et al. Changes in the chondrocyte and extracellular matrix proteome during post-natal mouse cartilage development. **MOL. CELL PROTEOMICS** 2012;11(1):M111.014159.

12. Cecil DL, Johnson K, Rediske J, et al. Inflammation-induced chondrocyte hypertrophy is driven by receptor for advanced glycation end products. **J. IMMUNOL.** 2005;175(12):8296–8302.
